# An IL‐12‐Based Nanocytokine Safely Potentiates Anticancer Immunity through Spatiotemporal Control of Inflammation to Eradicate Advanced Cold Tumors

**DOI:** 10.1002/advs.202205139

**Published:** 2023-02-05

**Authors:** Pengwen Chen, Wenqian Yang, Koji Nagaoka, George Lo Huang, Takuya Miyazaki, Taehun Hong, Shangwei Li, Kazunori Igarashi, Kazuyoshi Takeda, Kazuhiro Kakimi, Kazunori Kataoka, Horacio Cabral

**Affiliations:** ^1^ Department of Bioengineering Graduate School of Engineering The University of Tokyo 7‐3‐1 Hongo Bunkyo‐ku Tokyo 113‐8656 Japan; ^2^ Department of Immunotherapeutics The University of Tokyo Hospital 7‐3‐1 Hongo, Bunkyo‐ku Tokyo 113‐8655 Japan; ^3^ Red Arrow Therapeutics, Inc. 7‐3‐1 Hongo, Bunkyo‐ku Tokyo 113‐0003 Japan; ^4^ Kanagawa Institute of Industrial Science and Technology 705‐1Shimoimaizumi Ebina City Kanagawa 243‐0435 Japan; ^5^ Department of Otorhinolaryngology and Head and Neck Surgery Graduate School of Medicine and Faculty of Medicine The University of Tokyo 7‐3‐1 Hongo, Bunkyo‐ku Tokyo 113‐0033 Japan; ^6^ Department of Biofunctional Microbiota Graduate School of Medicine Juntendo University 2‐1‐1 Hongo, Bunkyo‐ku Tokyo 113‐8421 Japan; ^7^ Laboratory of Cell Biology Research Support Center Graduate School of Medicine Juntendo University 2‐1‐1 Hongo, Bunkyo‐ku Tokyo 113‐8421 Japan; ^8^ Innovation Center of NanoMedicine (iCONM) Kawasaki Institute of Industrial Promotion 3‐25‐14 Tonomachi, Kawasaki‐ku Kawasaki 210‐0821 Japan

**Keywords:** breast cancer, cytokine, immune checkpoint inhibitor, immunotherapy, interleukin‐12, metastatic tumor

## Abstract

Treatment of immunologically cold tumors is a major challenge for immune checkpoint inhibitors (ICIs). Interleukin 12 (IL‐12) can invigorate ICIs against cold tumors by establishing a robust antitumor immunity. However, its toxicity and systemic induction of counteracting immunosuppressive signals have hindered translation. Here, IL‐12 activity is spatiotemporally controlled for safely boosting efficacy without the stimulation of interfering immune responses by generating a nanocytokine that remains inactive at physiological pH, but unleashes its full activity at acidic tumor pH. The IL‐12‐based nanocytokine (Nano‐IL‐12) accumulate and release IL‐12 in tumor tissues, eliciting localized antitumoral inflammation, while preventing systemic immune response, counteractive immune reactions, and adverse toxicities even after repeated intravenous administration. The Nano‐IL‐12‐mediated spatiotemporal control of inflammation prompt superior anticancer efficacy, and synergize with ICIs to profoundly inflame the tumor microenvironment and completely eradicate ICI‐resistant primary and metastatic tumors. The strategy could be a promising approach toward safer and more effective immunotherapies.

## Graphical abstract

An interleukin 12 (IL‐12)‐based nanocytokine is formulated by a pH‐sensitive polymeric coating strategy. The nanocytokine can spatiotemporally control the inflammation induced by IL‐12, enhancing the antitumoral immunity without initiating systemic response. The nanocytokine can serve as monotherapy and synergize with checkpoint inhibitors to completely eradicate immunosuppressive primary and metastatic tumors without showing side effects.

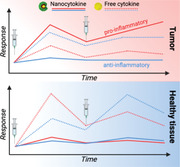

## 1 Introduction

Immune checkpoint inhibitors (ICIs), such as anti‐PD‐1 (nivolumab) and anti‐CTLA‐4 (ipilimumab) antibodies, have revolutionized cancer therapy.^[^ ^1^, ^2^ ^]^ However, a large number of patients still do not benefit from these treatments.^[^ ^3^ ^]^ Such deficient response has been associated with a cold tumor phenotype that is insensitive to ICIs.^[^ ^4^, ^5^ ^]^ These cold tumors usually present an immunosuppressive microenvironment and low levels of T‐cell infiltration that weakens the effects of the ICIs.^[^ ^5^, ^6^, ^7^ ^]^ Thus, there is an urgent need for agents shifting the tumor from the cold phenotype into an inflamed (hot) phenotype with high T‐cell infiltration toward enhancing the efficacy of ICIs and expanding the therapeutic landscape.

Interleukin‐12 (IL‐12), which is among the strongest proinflammatory cytokines, has high potential for boosting antitumor immunity and overcoming ICI resistance.^[^ ^8^, ^9^, ^10^ ^]^ However, IL‐12 induces severe immune‐related adverse events (irAEs) when systemically injected.^[^ ^8^, ^10^ ^]^ Thus, a variety of protein engineering approaches, including immunocytokines,^[^ ^11^, ^12^, ^13^ ^]^ fusion proteins,^[^ ^14^ ^]^ and protease‐sensitive cytokines,^[^ ^15^ ^]^ are under intense investigation for improving the safety of IL‐12 by reducing systemic exposure and enhancing tumor selectivity. On the other hand, IL‐12 still presents critical drawbacks related to the spatiotemporal dynamics of the inflammation that lead to counteractive immune reactions undermining its efficacy.^[^ ^16^, ^17^, ^18^, ^19^ ^]^ For example, repeated IL‐12 injection promoted the systemic expansion of anti‐inflammatory interleukin‐10 (IL‐10) in patients, which limited the anti‐tumor efficacy.^[^ ^20^, ^21^, ^22^ ^]^ In this regard, developing systems capable of spatiotemporally controlling the IL‐12‐mediated inflammation could maximize the effector functions and stimulate a robust antitumor immunity, while avoiding interfering immune reactions. Unfortunately, the inflammatory dynamics for the abovementioned protein‐engineered systems are not fully understood, and their association with counteracting secondary responses remains to be clarified.

Herein, we developed stimuli‐responsive nanoscaled cytokines (nanocytokines) by encapsulating native IL‐12 with biocompatible polymers to attain spatiotemporal control of its activity. The IL‐12‐based nanocytokines (Nano‐IL‐12) were designed to unleash the fully active IL‐12 after sensing the acidic intratumoral pH, as acidosis is a hallmark of cancer^[^ ^23^ ^]^ and is linked with immunosuppression.^[^ ^24^, ^25^ ^]^ In fact, we have recently found that the pH‐switchable function can allow high and selective activation in ICI‐resistant tumors.^[^ ^26^ ^]^ The pharmacokinetics, safety, and efficacy of the Nano‐IL‐12 were evaluated in murine models of cold melanoma and breast cancer. Our results showed Nano‐IL‐12 induces a sustained inflammatory reaction in tumor tissues by increasing the intensity and duration of the exposure to IL‐12, while avoiding the interaction with the immune cells in healthy tissues to suppress the off‐target immune response. Thus, Nano‐IL‐12 effectively blocked counteracting secondary reactions both systemically and intratumorally, showing efficacy at doses that are 10‐fold lower than native IL‐12. The enhanced antitumor immunity of Nano‐IL‐12 safely synergized with ICIs to intensely inflame the tumor microenvironment (TME), leading to complete responses (CR) in ICI‐resistant models of melanoma, and primary and lung metastasis of triple‐negative breast cancer (TNBC).

## 2 Results

### 2.1 Nano‐IL‐12 Senses Intratumoral pH to Release Fully Active Cytokine

**Figure 1:**
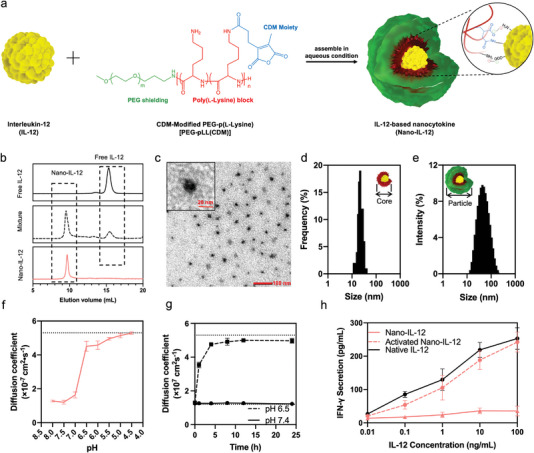
Nano‐IL‐12 activates at intratumoral pH to release the fully active cytokine. a) Formation and structure of IL‐12‐based nanocytokine (Nano‐IL‐12). The Nano‐IL‐12 is self‐assembled in aqueous conditions by simply mixing the polymer with IL‐12. The assembly is driven by the pH‐sensitive amide bonds and electrostatic interactions. b) HPLC results of free IL‐12 protein, PEG‐pLL(CDM) + IL‐12 mixture, and purified Nano‐IL‐12. The IL‐12 proteins are labeled with A647. c) Representative TEM image of purified Nano‐IL‐12 clearly shows the core structure of the particles. d) Distribution of the particle cores diameter measured from the TEM images. *n* = 100 particles counted. e) Distribution of the hydrodynamic diameters of Nano‐IL‐12 measured by DLS. f) pH‐dependent Nano‐IL‐12 disassociation indicated by FCS measurement of Nano‐IL‐12 incubated under different pH for 24 h. The dotted line at 5.3 × 10^7^ cm^2^ s^−1^ refers the diffusion coefficient of free IL‐12. g) IL‐12 release profile of Nano‐IL‐12 measured by the FCS method. The dotted lines at 1.2 × 10^7^ cm^2^ s^−1^ and 5.3 × 10^7^ cm^2^ s^−1^ refer to the diffusion coefficients of intact Nano‐IL‐12 and released free IL‐12, respectively. h) IFN‐*γ* secretion by murine splenocytes treated with Nano‐IL‐12, activated Nano‐IL‐12 and native IL‐12. IFN‐*γ* was measured by ELISA. Data are shown as mean ± S.D.; for f and g, *n* = 3 parallel measurements. For h, *n* = 5 parallel measurements.

To construct the Nano‐IL‐12, we synthesized carboxydimethyl‐maleic anhydride (CDM)‐modified poly(ethylene glycol)‐poly(L‐Lysine) (PEG‐pLL(CDM)) through the reported method^[^ ^27^ ^]^ (Scheme S1, Supporting Information). The polymer has half of the amino groups in the pLL block conjugated with CDM through the amide‐linkage (Figures S1–S3, Supporting Information). The Nano‐IL‐12 are formed just by mixing the polymer with the cytokine in aqueous condition without the addition of organic solvents (**Figure** **1**a). The amino groups in the polymer form ion complexes with the carboxylate moieties in the proteins, while the CDM groups react with the amino groups in IL‐12 as well as in the polymer strands to form pH‐sensitive amide bonds. The encapsulation efficiency of IL‐12 in the nanocytokines was determined to be around 80%, as measured by HPLC by comparing the areas of the peaks of Alexa Fluor 647 (A647)‐labeled free IL‐12 and the IL‐12 loaded in the nanocytokine (Figure 1b). In the case of non‐fluorescence labeled IL‐12, the encapsulation efficiency was validated by ELISA measurement, since the ELISA method can only detect the unencapsulated free IL‐12 in the reacted mixture because the polymer coating of Nano‐IL‐12 blocks the recognition of the loaded IL‐12 with the detection antibody. Thus, by calculating the ratio of the unencapsulated IL‐12 versus the total IL‐12 feed, the encapsulation efficiency was also determined to be around 80% (Figure S4a, Supporting Information), consistent with the result from the HPLC measurements. The Nano‐IL‐12 were purified by centrifugal filtration to remove free IL‐12 and relatively small polymer‐IL‐12 conjugates (Figure S4b, Supporting Information). The purification was confirmed by HPLC (Figure 1b, lower panel).

The formation of the polymeric shield on Nano‐IL‐12 was confirmed by transmission electron microscopy (TEM) and dynamic light scattering (DLS). In TEM, the IL‐12‐loaded core of the Nano‐IL‐12 formed by the pLL block of the polymer and IL‐12 were stained by uranyl acetate^[^ ^28^ ^]^ and visualized as uniform black dots with average diameter at 23.2 ± 4 nm (Figure 1c,d). By DLS, the hydrodynamic diameter of the Nano‐IL‐12 was measured to be 42 ± 2 nm (Figure 1e). By comparing the size measured by TEM and DLS, we can calculate that the thickness of the PEG shell in these particles is approximately 10 nm. This result indicates a dense PEGylation, which would be useful for improving the pharmacokinetics.^[^ ^29^ ^]^ Moreover, fluorescence correlation spectroscopy (FCS) studies of the A647‐labeled Nano‐IL‐12 confirmed the formation of associates with larger molecular weight than free A647‐labeled IL‐12 (Table S1, Supporting Information). By comparing the counts per molecule in the FCS measurement of A647‐labeled IL‐12 and A647‐labeled Nano‐IL‐12 samples, we determined that each Nano‐IL‐12 contains approximately 1.5 IL‐12 molecules in average. In addition, the surface charge of the Nano‐IL‐12 was found to be close to neutral (Table S2, Supporting Information), suggesting the polymer is shielding the negatively charged IL‐12.

The amide bonds in the Nano‐IL‐12 formed between the CDM moieties and primary amines are reported to be pH‐sensitive.^[^ ^30^ ^]^ Thus, the Nano‐IL‐12 are expected to dissociate under acidic conditions to release the encapsulated cytokine. We first screened the pH‐sensitivity of the Nano‐IL‐12 at various pH values ranging from pH 4.5 to 8.5 by FCS measurement to reflect the dissociation status of the Nano‐IL‐12 by the change of the diffusion coefficient. The increase of the diffusion coefficient, corresponding to the decrease of the molecular weight of the particles, could be observed under acidic condition below pH 7.0, suggesting that the Nano‐IL‐12 are stable at physiological pH, but could lose their integrity in intratumoral lesions with the acidified environment^[^ ^23^, ^31^ ^]^ (Figure 1f). Next, we investigated the dissociation kinetics of Nano‐IL‐12 at physiological pH 7.4 and intratumoral pH 6.5 by FCS measurement. The FCS measurement allows precise assessment of the diffusion coefficients of the Nano‐IL‐12 and the released IL‐12. The new result showed the Nano‐IL‐12 rapidly lost integrity at pH 6.5, and the diffusion coefficient of the sample reached the value of free IL‐12 after 4 h incubation, suggesting complete activation of Nano‐IL‐12 under such conditions. Moreover, at pH 7.4, the Nano‐IL‐12 were stable (Figure 1g), retaining their diffusion coefficient even after several days (Figure S4c, Supporting Information). These observations support the selective activation of Nano‐IL‐12 at intratumoral pH conditions and the stability at physiological conditions.

Since IL‐12 can stimulate the secretion of IFN‐*γ* from splenocytes,^[^ ^32^ ^]^ the effect of polymer shielding/de‐shielding on the bioactivity of Nano‐IL‐12 was evaluated in vitro by splenocyte assay (Figure 1h). The Nano‐IL‐12 induced lower levels of IFN‐*γ* production from murine splenocytes, indicating the blockage of IL‐12 bioactivity by the polymer encapsulation. On the other hand, the Nano‐IL‐12 activated by pre‐incubation in acid (pH 6.5) showed similar bioactivity to that of native IL‐12, suggesting the activated Nano‐IL‐12 can fully retrieve the bioactivity of the cytokine.

The preservation and stability of the formulation are also important for translating Nano‐IL‐12 as a drug. Therefore, we developed a lyophilized version of Nano‐IL‐12 using trehalose as the cryoprotectant.^[^ ^33^ ^]^ The reconstituted Nano‐IL‐12 showed comparable size and surface charge with fresh Nano‐IL‐12, without cargo leakage, and shared comparable pH sensitivity and in vitro IFN‐*γ* inducement capability (Figure S4d–g and Table S2, Supporting Information). These results support the lyophilized formulation as a viable Nano‐IL‐12 counterpart.

### 2.2 Nano‐IL‐12 Improves IL‐12 Pharmacokinetics and Activates in Tumors to Potentiate Antitumor Effects

**Figure 2:**
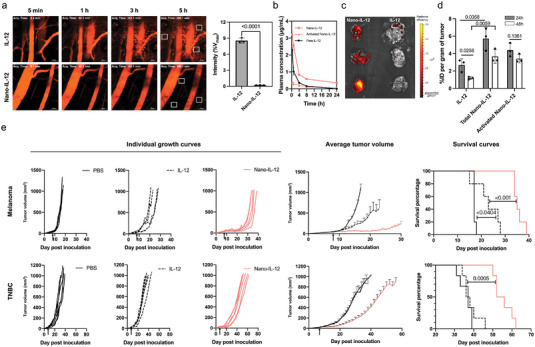
Nano‐IL‐12 improves pharmacokinetics and anti‐tumor efficacy. a) IVCLSM images of the earlobe skin of mice after i.v. injection of 10 µg A647‐labeled IL‐12 or Nano‐IL‐12 (red color). Scale bar = 50 µm. Mean fluorescence intensity in the tissue area (white boxes) at 5 h after injection were quantified and normalized to the maximum intensity in the vasculature immediately after injection (*V*_max_). b) Blood circulation profiles of free IL‐12 and Nano‐IL‐12 after i.v. injection of 10 µg IL‐12 or equivalent Nano‐IL‐12 determined by ELISA. Also, the concentration of released IL‐12 from Nano‐IL‐12 in blood is plotted (data are shown as mean ± S.D., *n* = 5 mice per group). c) IVIS image of B16F10 melanoma tumors excised 24 h post i.v. injection of 10 µg IL‐12 or equivalent Nano‐IL‐12 labeled with A647. d) Quantification of the IL‐12 level in 4T1 TNBC tumors at 24‐ and 48 h post i.v. injection of 10 µg IL‐12 or equivalent Nano‐IL‐12 by ELISA (Data are shown as mean ± S.D.; *n* = 3 mice per group; *p* values are calculated by one‐way ANOVA). e) Anti‐tumor activity of a single i.v. injection (injection days are indicated by the arrow (Day 8 for B16F10 model and Day 7 for 4T1 model)) of 10 µg IL‐12 or equivalent Nano‐IL‐12. The results in B16F10 melanoma are shown in the upper panel and the results in the 4T1 TNBC are shown in the lower panel. The individual tumor growth curves are shown in the left panels. The average tumor volumes curves are shown in the center panels, and the survival curves are shown in the right panel (Data are shown as mean ± SEM; *n* = 5 mice per group, *p* values are calculated via log‐rank analysis).

The encapsulation into the Nano‐IL‐12 can improve the pharmacokinetics of IL‐12. For visualizing the circulation of Nano‐IL‐12 upon intravenous (i.v.) injection, in vivo confocal laser scanning microscopy (IVCLSM) was used to track the A647‐IL‐12‐based Nano‐IL‐12 in the vessels of mouse earlobes after injection from the tail vein. Free IL‐12 showed extravasation after injection (**Figure** **2**a), indicated by the increased fluorescence intensity in tissue interstitium. Considering that macromolecules having a comparable size to IL‐12 (75 kDa), such as 70 kDa dextran and albumin (65 kDa), show limited access into skin,^[^ ^34^ ^]^ the leakage of IL‐12 from vessels suggests the instability of IL‐12 during blood circulation. In fact, previous studies have indicated the proteolysis of IL‐12 by enzymes present in serum.^[^ ^35^, ^36^ ^]^ To further support the degradation of IL‐12 in our experiments, the stability of A647‐labeled IL‐12 in mouse plasma was investigated by size exclusion chromatography (SEC). Upon incubation with mouse plasma, we found that IL‐12 degraded into fragments with smaller molecular weight (Figure S5, Supporting Information), confirming the instability of IL‐12 in blood. On the other hand, the Nano‐IL‐12 showed minimal leakage into the skin, indicating their stability in blood circulation. The quantified circulation profile measured from ELISA assay confirmed the overall Nano‐IL‐12 has longer circulation than free IL‐12 (Figure 2b). Meanwhile, the level of activated Nano‐IL‐12, which was determined by the released IL‐12, was highly restricted in blood, indicating the in vivo stability of Nano‐IL‐12 during systemic circulation, which helps to reduce the systemic side effects.

The accumulation of Nano‐IL‐12 in heart, spleen, lung and kidneys was similar to that of free IL‐12 at both 24 and 48 h after administration, while in liver, the accumulation of Nano‐IL‐12 was higher than that of free IL‐12 at 24 h, but comparable at 48 h (Figure S6, Supporting Information). Nano‐IL‐12 showed significantly higher tumor accumulation than free IL‐12 (Figure 2c) at 24 h after injection. Moreover, while free IL‐12 was cleared from the tumors at 48 h after injection, the concentration of Nano‐IL‐12 remained high (around 4‐fold higher than free IL‐12) (Figure 2d), suggesting Nano‐IL‐12 increased the area under the concentration curve (AUC) of IL‐12 inside the tumors. In line with the elevated accumulation and viable activation in tumor, Nano‐IL‐12 showed improved antitumoral efficacy over free IL‐12 in subcutaneous B16F10 melanoma and orthotopic 4T1 triple‐negative breast cancer (TNBC) models (Figure 2e). In both models, just a single i.v. injection of Nano‐IL‐12 at 10 µg IL‐12 equivalent led to a significantly improved inhibition of tumor growth than IL‐12, leading to longer survival time. Notably, in 4T1 TNBC model, which is infamous for its strong immunosuppression and resistance against ICI treatment,^[^ ^37^ ^]^ the treatment with Nano‐IL‐12 resulted in a clearly significant therapeutic effect, whereas free IL‐12 was futile against tumor growth.

### 2.3 Nano‐IL‐12 Spatiotemporally Controlled the Inflammatory Response

To further investigate the difference between the biological effects of Nano‐IL‐12 and free IL‐12, the level of inflammation response evoked from the treatment was evaluated by the cytokine response both in off‐target sites, i.e., blood and healthy organs, and in tumors (orthotopic 4T1 model). Four down‐stream cytokines of IL‐12, namely, IFN‐*γ*, tumor necrosis factor‐*α* (TNF‐*α*), interleukin‐6 (IL‐6), and interleukin‐10 (IL‐10) were selected as indicators of inflammation response. Among them, IFN‐*γ*, TNF‐*α*, and IL‐6 are important proinflammatory cytokines associated with the antitumoral effect of IL‐12,^[^ ^20^, ^38^ ^]^ while IL‐10 is an anti‐inflammatory cytokine, which is a negative feedback antagonizing IL‐12 stimulation.^[^ ^20^, ^39^ ^]^

**Figure 3:**
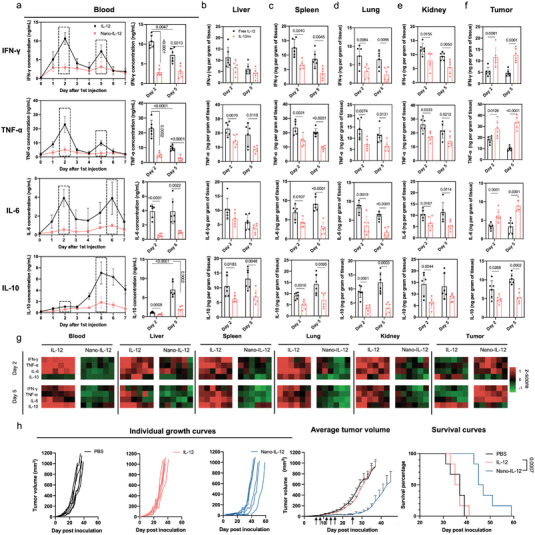
Nano‐IL‐12 spatiotemporally controlled the inflammatory response. a) Proinflammatory (IFN‐*γ*, TNF‐*α*, IL‐6) and anti‐inflammatory (IL‐10) cytokine levels in blood in 4T1‐bearing mice injected with 10 µg IL‐12 or equivalent Nano‐IL‐12 twice on Days 0 and 3. The cytokine levels were measured by ELISA. The peak values after the two injections (on Days 2 and 5 for IFN‐*γ*, TNF‐*α* and IL‐10; on Days 2 and 6 for IL‐6) are visualized as bar graphs on the right panel. b–f) Cytokine levels in the organs and tumors of 4T1‐bearing mice injected with 10 µg IL‐12 or equivalent Nano‐IL‐12 twice on Days 0 and 3. The mice were sacrificed on Days 2 and 5 to collect the tissues. (Data are shown as mean ± S.D.; *n* = 6 mice per group; *p* values are calculated via unpaired t‐test.) g) Heatmaps of the cytokine levels in blood, organs, and tumors from the average values in a‐f converted to Z‐score. h) Anti‐tumor activity of repeated i.v. injection (injected on Days 7, 9, 11, 13, 15 and 25, indicated by arrows) of 1 µg IL‐12 or equivalent Nano‐IL‐12 against murine TNBC. The individual tumor growth curves are shown in the left panel. The average tumor volumes curves are shown in the central panel, and the survival curves are shown in the right panel (Data are shown as mean ± SEM; *n* = 6 mice per group, *p* values are calculated via log‐rank analysis).

Nano‐IL‐12 and free IL‐12 were injected two times on Days 0 and 3, and the inflammation response was tracked daily for 1 week. This experimental setting allowed us to determine the effects of both single injection and repeated dosing on the spatiotemporal inflammatory response. The first injection of free IL‐12 on Day 0 led to the secretion of the four cytokines in blood and organs, with a peak of plasma concentration observed on Day 2 (**Figure** **3**a–e,g), indicating the strong off‐target inflammation associated with the immune‐related adverse events (irAEs) of IL‐12.^[^ ^8^, ^10^ ^]^ On the other hand, the first injection of Nano‐IL‐12 showed significantly lower cytokine levels than free IL‐12 in blood and healthy organs, indicating the regulation of the off‐target inflammation response. The second injection of free IL‐12 on Day 3 again stimulated the secretion of IFN‐*γ*, TNF‐*α*, and IL‐6. However, the peak levels of IFN‐*γ* and TNF‐*α* observed on Day 5 were clearly lower than those observed on Day 2 after the first IL‐12 injection. Moreover, the second injection of free IL‐12 extremely raised the level of anti‐inflammatory IL‐10 in plasma, organs, and tumors, which has been associated with the systemic hyper‐expansion of Th1 cells to induce a regulatory phase.^[^ ^40^ ^]^ Such differentiated response induced from repetitive IL‐12 treatment has been confirmed in human clinical trials, and it contributes to the reduction of the biological effects of IL‐12, including the antitumoral potency.^[^ ^20^ ^]^ Contrary to IL‐12, the second injection of Nano‐IL‐12 did not exacerbate the secretion of IL‐10 in blood and organs. Also, the concentration of the inflammatory cytokines in blood and healthy tissues was significantly lower than that of free IL‐12. These results suggest that Nano‐IL‐12 could not only reduce the systemic inflammatory response, but also overcome the limitations of repeated IL‐12 administration, which induced the counteractive anti‐inflammatory response.

In tumors, a single i.v. shot of Nano‐IL‐12 boosted the production of inflammatory IFN‐*γ*, TNF‐*α* and IL‐6, achieving significantly higher intratumoral concentrations than free IL‐12 treatment (Figure 3f,g). Meanwhile, the Nano‐IL‐12 treatment induced lower anti‐inflammatory IL‐10 in the tumors than free IL‐12, avoiding the onset of counter anti‐inflammatory response. Moreover, upon the second injection of Nano‐IL‐12, the high level of the inflammatory cytokines was maintained in the tumors, while the intratumoral IL‐10 concentration was kept low. This upregulation of the IFN‐*γ*, TNF‐*α*, and IL‐6 and downregulation of IL‐10 in tumors correlates with the high and sustained intratumoral levels of Nano‐IL‐12, and support an elevated inflammatory reaction for Nano‐IL‐12, which is critical for enhancing the antitumoral efficacy.^[^ ^41^ ^]^

To test if Nano‐IL‐12 could improve the efficacy through its spatiotemporal control of inflammation, we repeated the experiment of Figure 2e, but this time we treated the mice with a 10‐fold lower dose, i.e., 1 µg of IL‐12 equivalent, using a repeated dosing schedule that triggers the counteracting immune response for free IL‐12. Under this recurring low‐dose treatment, Nano‐IL‐12 clearly enhanced the anti‐tumor efficacy over IL‐12 in 4T1 TNBC tumors (Figure 3h), leading to slower tumor growth and prolonged survival. On the other hand, the free IL‐12 treatment showed negligible efficacy even after being intensively injected for six times, which could be attributed to the expansion of anti‐inflammatory cytokines like IL‐10. These results support the ability of Nano‐IL‐12 to potentiate the antitumor efficacy by spatiotemporally controlling inflammation.

The control of the inflammatory response also led to enhanced safety. We conducted a histological evaluation and blood analysis to determine the toxicity of IL‐12 and Nano‐IL‐12 after two injections on Days 0 and 3 (Figure S7a,c, Supporting Information). The results showed significantly lower toxicity to liver, kidney and pancreas upon Nano‐IL‐12 treatment compared to free IL‐12. Also, the bodyweight changes during the treatments were tracked as a parameter of toxicity. Nano‐IL‐12 treated animals slightly gained weight during the treatment, whereas the mice receiving free IL‐12 suffered from bodyweight decrease (Figure S7b, Supporting Information). Thus, these observations indicate Nano‐IL‐12 can preferentially promote the inflammation response in tumors to enhance the antitumoral efficacy, while restricting off‐target effects in healthy tissues to reduce the toxicity and diminish the counteractive anti‐inflammatory response.

### 2.4 Nano‐IL‐12 Treatment Induces Antitumoral Immune Cell Infiltration in the TME

**Figure 4:**
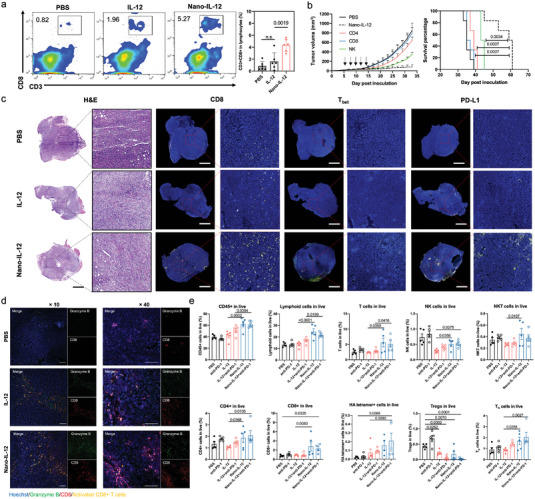
The anti‐tumor effect of Nano‐IL‐12 is generated from the enhanced infiltration of effector cells in the TME. a) Flow cytometry analysis of CTLs infiltration in melanoma after Nano‐IL‐12 treatment. Mice were inoculated with B16F10 cells on Day 0. IL‐12 (1 µg) or equivalent Nano‐IL‐12 were i.v. injected twice on Days 8 and 11. Tumor samples were collected and analyzed on Day 15 (Data shown as mean ± S.D.; *n* = 5 mice per group; *p*‐values were calculated via one‐way ANOVA). b) Antitumor activity of Nano‐IL‐12 upon depletion of CD4^+^, CD8^+^ and NK cells. Mice bearing 4T1 tumors were i.v. injected with Nano‐IL‐12 (1 µg IL‐12 equivalent) on Days 7, 9, 11, 13, and 15. Moreover, the mice were i.p. injected with anti‐CD4, anti‐CD8 and anti‐asialo GM1 antibodies on Days 6, 8, and 10. Tumor growth curves (Data are shown as mean ± SEM) and survive curves were recorded (*n* = 6 mice per group, *p* values are calculated via log‐rank analysis). c) IHC images of 4T1 tumor sections. Mice bearing 4T1 TNBC tumors (average tumor volume: 200 mm^3^) were twice i.v. injected with PBS, 10 µg IL‐12 or equivalent Nano‐IL‐12 on Days 0 and 3. On Day 7, the mice were sacrificed for collecting the tumors. The CD8^+^, T_bet_ or PD‐L1^+^ cells in the tumors are visualized in yellow. The cell nuclei were stained with Hoechst (blue). Scale bar = 1 mm. d) Immunostaining of Granzyme B (green) and CD8 (red) in 4T1 tumor sections. The cell nuclei were stained with Hoechst (blue). Yellow pixels indicate to activated CD8^+^ T cells. Scale bar = 100 µm. e) Analysis of lymphoid cells infiltration in 4T1‐HA tumors treated with PBS, anti‐PD1 antibodies, IL‐12, Nano‐IL‐12, IL‐12 plus anti‐PD1 antibodies and Nano‐IL‐12 plus anti‐PD1 antibodies. IL‐12 and Nano‐IL‐12 were i.v. injected at 10 µg on Days 7 and 9 postinoculation. Anti‐PD‐1 were i.p. injected at 100 µg on Days 8 and 10 postinoculation. On Day 17, mice were sacrificed, and the tumors were homogenized for flow cytometry measurement (Data are shown as mean ± S.D., *n* = 5 samples per group, *p* values are calculated via one‐way ANOVA).

IL‐12 is also known as T cell‐stimulating factor, as it can stimulate the growth and function of T cells to initiate the enhanced antitumoral immune reactions. Thus, we evaluated the infiltration of CD8^+^ cells in tumors after repeated Nano‐IL‐12 treatment. Flow cytometry analysis of the B16F10 melanoma treated by two i.v. injections of free IL‐12 or Nano‐IL‐12 at 1 µg IL‐12 equivalence/injection showed that Nano‐IL‐12 significantly enhanced the infiltration of CD8^+^ T cells in the tumor compared to free IL‐12 (**Figure** **4**a). By doing an immune cell depletion experiment in mouse bearing 4T1 TNBC tumors, we investigated the contribution of different immune cell populations to the response from Nano‐IL‐12 treatment. Nano‐IL‐12 were i.v. injected five times on Days 7, 9, 11, 13, and 15 (1 µg IL‐12 equivalence/injection). Moreover, the mice were intraperitoneally (i.p.) injected with anti‐CD4, anti‐CD8 or anti‐asialo GM1 antibodies on Days 6, 8, and 10 to deplete CD4^+^, CD8^+^ or NK cells respectively. The results showed the depletion of CD8^+^ cytotoxic T cells, CD4^+^ T cells and NK cells significantly decreased the therapeutic efficacy of Nano‐IL‐12, as indicated by the faster tumor growth and the decreased survival in the depleted groups compared to the mice receiving only the Nano‐IL‐12 treatment (Figure 4b). This result is consistent with the biological function of IL‐12, including enhancing the cytotoxic activity of CD8 T cells^[^ ^42^ ^]^ and NK cells,^[^ ^43^ ^]^ and mediating the differentiation of naïve T cells into T helper (T_h_) cells.^[^ ^44^ ^]^ Moreover, the different mitigation efficiency of the antitumor activity resulting from the depletion experiment suggests the relevance of each cell population on the efficacy of Nano‐IL‐12, with CD8^+^ cytotoxic T cells being the most important fraction.

Immunohistochemistry analysis of 4T1 TNBC tumor sections revealed the spatial distribution of tumor‐infiltrating effector cells (Figure 4c). While free IL‐12 did not improve the presence of CD8^+^ cytotoxic T cells and T_bet_+ (T_h1_) cells in the tumors, the Nano‐IL‐12 promoted high infiltration of both cells to deep tumor regions. Also, the upregulation of Granzyme B in CD8^+^ Cells suggested a higher activation level of cytotoxic T cells after Nano‐IL‐12 treatment (Figure 4d). Moreover, we observed Nano‐IL‐12 treatment elevated the PD‐L1 expression in tumors compared to tumors treated by PBS and free IL‐12. This increased PD‐L1 level could be due to the higher tumor IFN‐*γ* induced by Nano‐IL‐12, which have been reported to stimulate PD‐L1 expression in tumor tissue,^[^ ^45^ ^]^ serving as a mechanism of immunosuppression to resist lymphocyte infiltration. This intratumoral PD‐L1 upregulation suggests the potential to synergize the antitumor efficacy of Nano‐IL‐12 by combining it with anti‐PD‐1 or anti‐PD‐L1 ICIs.

Based on the observation above, we investigated the effect of Nano‐IL‐12 as a monotherapy and in combination with anti‐PD‐1 antibodies on the TME (Figure 4e). The experiment was done in orthotopic TNBC tumors prepared from 4T1 cells expressing hemagglutinin (4T1‐HA), since hemagglutinin is a well‐defined immunogen, which can induce antigen‐specific cytotoxic T‐lymphocyte response in mouse model.^[^ ^46^, ^47^ ^]^ The mice were twice treated with 10 µg IL‐12 or equivalent Nano‐IL‐12 on Day 0 and 3, and for combination therapy the mice simultaneously received twice injections of 100 µg anti‐PD‐1 antibody. On Day 7, the tumor samples were harvested for flow‐cytometry. The results confirmed Nano‐IL‐12 treatment induced higher infiltration of leukocytes (CD45^+^) in the tumor than IL‐12. The combination of Nano‐IL‐12 with the anti‐PD‐1antibodies also presented higher CD45^+^ cells in the tumors. Among leukocytes, lymphoid cells and T cells were upregulated by Nano‐IL‐12 and by the Nano‐IL‐12/anti‐PD‐1antibody combination. The Nano‐IL‐12 treatment resulted in a modest alteration on NK cell population, but led to significant changes on T cell subgroup compared to free IL‐12. Notably, the infiltration of CD8^+^ T cells in 4T1‐HA tumors was clearly elevated by Nano‐IL‐12 than free IL‐12. Moreover, the HA‐tetramer^+^ T cells, which correspond to the antigen‐specific T cells, were significantly increased in Nano‐IL‐12 plus anti‐PD‐1 antibody combination, suggesting the promotion of an adaptive immune response by the synergy of Nano‐IL‐12 and ICIs.

The general CD4^+^ T cells showed upregulation in the Nano‐IL‐12 treated groups. To furtherly specify the subpopulation of CD4^+^ T cells in the tumors, we stained the cells with anti‐Foxp3 for investigating the regulatory T cells (Tregs). While anti‐PD‐1 monotherapy did not show any suppression on Tregs in the 4T1‐HA tumor model, the combination of Nano‐IL‐12 plus anti‐PD‐1 dramatically depleted the Tregs. Moreover, Nano‐IL‐12 treated tumors presented higher T_h_ cells than free IL‐12. These results indicate Nano‐IL‐12 treatment enhanced anti‐tumor immune cell infiltration and synergized with anti‐PD‐1 antibodies to surmount the immunosuppressive TME.

### 2.5 Nano‐IL‐12 Treatment Activates the TME from the Transcription Level to Potentiate ICI Response

**Figure 5:**
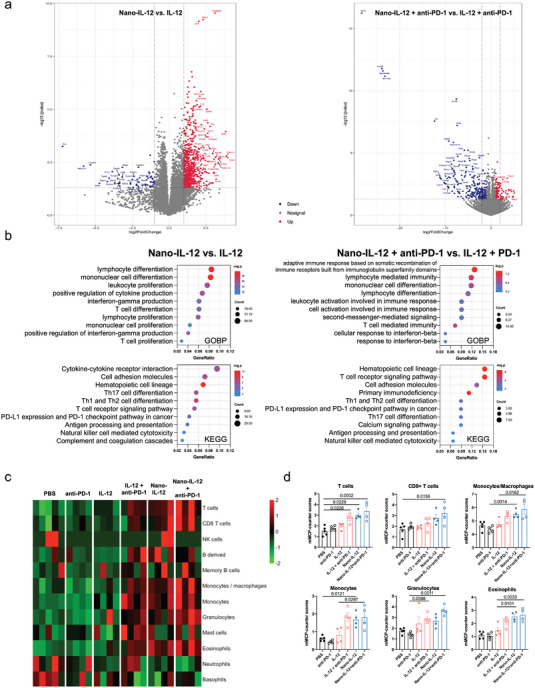
Nano‐IL‐12 treatment enhances immune activation in TME to potentiate anti‐PD1 antibodies. a) Volcano plots of the differentially expressed genes in comparison between Nano‐IL‐12 versus IL‐12 and Nano‐IL‐12 + anti‐PD‐1 versus IL‐12 + anti‐PD‐1. The plots were obtained from RNA‐seq analysis of 4T1‐HA tumors treated by different treatments: 10 µg IL‐12 and equivalent Nano‐IL‐12 were i.v. injected on Days 7 and 9 postinoculation. Anti‐PD‐1 were i.p. injected at 100 µg on Days 8 and 10 postinoculation. On Day 17, mice were sacrificed to collect the tumor samples. b) Enrichment analysis showing the upregulated pathways in the two comparison pairs in a. c) Heatmap of cell populations in the TME determined by RNA‐seq. The populations were converted to Z‐scores for plotting the figure. d) Histograms of the cell populations showing significant differences in multiple comparison (Data are shown as mean ± S.D.; for PBS group, *n* = 5 samples; for other groups, *n* = 4 samples per group; *p* values are calculated via one‐way ANOVA).

We then investigated the activation of the TME of 4T1‐HA tumors after the treatments from the gene expression level by transcriptome analysis. Sequencing of the total RNA samples extracted from tumor tissues was conducted after treatment with PBS, anti‐PD‐1 antibodies, IL‐12, Nano‐IL‐12 and the combinations of the cytokines with ICIs. The heatmap of global gene expression clearly indicated the differentiated gene profiles in tumors receiving Nano‐IL‐12 and the combination of Nano‐IL‐12 with anti‐PD‐1 (Figure S11, Supporting Information). For specifying the affected biological processes from Nano‐IL‐12 treatment, we analyzed the differentially expressed genes in each group, and conducted an enrichment analysis on the upregulated and downregulated genes (**Figure** **5**a,b). Both GOBP and KEGG analysis revealed that, compared with IL‐12 and PBS, Nano‐IL‐12 treatment distinctly upregulated the stimulation of proliferation, differentiation, and functional activation of various effector immune cells, such as T cell proliferation, differentiation of T cells into T_h_ cells, and T cell activation and T cell receptor signaling pathway. Moreover, genes related to PD‐L1 expression and PD‐1 checkpoint pathway were also found to be upregulated upon Nano‐IL‐12 treatment. These results support the robust stimulation of Nano‐IL‐12 to T cells observed in the flow cytometry and immunohistology studies. At the same time, genes associated with other immune cells, like monocytes and NK cells, were positively affected by the Nano‐IL‐12 treatment. Also, cytokine response and other immune process, like antigen‐presentation, were upregulated from Nano‐IL‐12 treatment. On the other hand, Nano‐IL‐12 downregulated pathways associated with cell division, suggesting that the treatment could suppress the proliferation of tumor cells (Figure S11, Supporting Information). These results indicate the advantages of Nano‐IL‐12 on activating both the innate and adaptive arm of the intratumoral immunity.

Further investigation on the combination therapies revealed that the combination treatment of Nano‐IL‐12 plus anti‐PD‐1 antibodies enhanced the intratumoral immunity compared to IL‐12 plus anti‐PD‐1 antibodies or anti‐PD‐1 monotherapy by stimulating both innate and adaptive immune pathways (Figure 5b and Figure S11c, Supporting Information). Impressively, the Nano‐IL‐12 plus anti‐PD‐1 antibodies combination resulted in stronger inhibition on the pathways associated with mitosis, inferring this combination could intermediate a stronger killing activity of the infiltrated effector cells against tumor cells.

Based on the RNA‐seq results, we also analyzed the populations of tumor‐infiltrating immune cells (Figure 5c,d). Like the result from flow cytometry, Nano‐IL‐12 treated groups showed enhanced infiltration of CD8^+^ T cells. Furthermore, the results also revealed the upregulation of monocytes and granulocytes, confirming the enhanced inflammation in tumor from the boosted innate immune pathways. These results confirmed Nano‐IL‐12 sets a potent immune response against tumors and the combination with anti‐PD‐1 can strengthen this process.

### 2.6 Nano‐IL‐12 Synergizes with ICIs to Effectively Eradicate Primary and Metastatic Tumors

**Figure 6:**
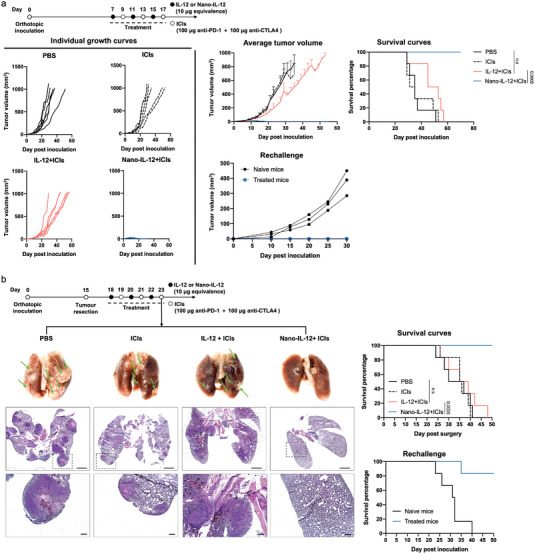
Nano‐IL‐12 synergizes with immune checkpoint inhibitors to eradicate breast tumors. In both orthotropic and metastatic TNBC model, mice were grouped to receive PBS, IL‐12 + ICIs (anti‐CTLA4 and anti‐PD‐1) and Nano‐IL‐12 + ICIs therapies (dose and treatment schedule were shown in the scheme at the upper panels respectively). a) Combination therapy of Nano‐IL‐12 and ICIs lead to complete eradication of orthotopic tumors in all mice treated. The individual tumor growth curves are shown in the left panel. The average tumor volume and survival curves are shown in the upper‐right panel. The cured mice showed robust defense against rechallenge injection with 4T1 cells to the mammary, with no tumor grew on the treated mice (lower‐right panel). b) Combination therapy of Nano‐IL‐12 and ICIs showed strong inhibition against metastatic tumor progression. After treatment (Day 23), the lungs of mice treated with Nano‐IL‐12 + ICIs combination therapy showed clearly fewer metastasis, shown by the representative photos (green arrows: macroscopic metastasis) and histological evaluation (Scale bar = 100 µm) presented in the left panel. Nano‐IL‐12 + ICIs combination therapy finally led to complete response in all mice treated, as indicated by the survival curves (upper‐right panel). Also, the cure mice showed robust defense against tumor rechallenge by injection of 4T1 cells (lower‐right panel). (Data are shown as mean ± SEM.; *n* = 6 mice per group; *p* values are calculated via log‐rank analysis).

To investigate the therapeutic potential of Nano‐IL‐12 to synergize with ICIs, orthotopic primary TNBC tumors were established in mice by inoculating 4T1 cells to the mammary fat pad. The mice were treated with Nano‐IL‐12 upon different dose schedules and combination patterns with ICIs to test the antitumoral efficacy of the corresponding treatments. We found that, even under at a low dose (1 µg IL‐12 equivalence/injection), the repeated treatment with Nano‐IL‐12 exerted a clear anti‐tumor activity against primary TNBC tumors as a monotherapy, as shown by the suppression of the tumor growth rate and the prolonged survival (Figure S12, Supporting Information). However, free IL‐12 treatment under this dose did not show any efficacy, even combined with anti‐PD‐1 therapy. The combination of Nano‐IL‐12 with anti‐PD‐1 further increased the efficacy, and CR was achieved. We then studied a higher Nano‐IL‐12 dose (10 µg IL‐12 equivalence/injection) and combined it with ICI cocktails (anti‐PD‐1 and anti‐CTLA4 antibodies) to fulfill the therapeutic potential. Notably, at this dose, the combination therapy of Nano‐IL‐12 with ICIs stopped the tumor growth and eradicated tumors completely (**Figure** **6**a), with all mice showing CR in this treatment group. Moreover, the cured mice showed resistance against tumor rechallenge with 4T1 cells, supporting the existence of a robust immune memory from the treatment with Nano‐IL‐12 and ICIs combination therapy.

TNBC is known as an aggressive tumor with high rate of distant metastasis.^[^ ^48^, ^49^ ^]^ Thus, we next investigated the performance of Nano‐IL‐12 in a metastatic TNBC model. A spontaneous metastatic TNBC model was established by resection the primary orthotopic 4T1 tumors, which will lead to the development of metastasis in multiple organs, mainly lungs.^[^ ^50^ ^]^ In this model, the combination therapy of Nano‐IL‐12 with ICIs also revealed satisfactory outcomes, which stopped the progression of lung metastasis (Figure 6b) and led to CR in all the treated mice. Moreover, upon rechallenge with an i.v. injection of 4T1 cells, most cured mice showed robust resistance, indicating an effective immune memory from the Nano‐IL‐12 and ICIs combination.

Besides TNBC, we also tested Nano‐IL‐12 in combination with ICI in a primary B16F10 melanoma model (Figure S13, Supporting Information). The results showed that the combination of Nano‐IL‐12 (10 µg IL‐12 equivalence/injection) with anti‐PD‐1 antibody led to CR in 4 mice out of 6 in the group, and the cured mice showed resistance against rechallenge with B16F10 cells, suggesting a strong immunological memory.

## 3 Discussion

We have developed a tumor‐activatable IL‐12 strategy using pH‐sensitive nanocytokines (Nano‐IL‐12) that can mute the bioactivity of IL‐12 at pH 7.4, but retrieve the fully active cytokine at intratumoral pH. Upon systemic injection, the Nano‐IL‐12 stably circulated in the bloodstream, reducing the immune response in non‐pathological sites and the incidence of irAEs, even after repeated administration. On the other hand, the high accumulation and activation of Nano‐IL‐12 in tumors lead to a significant enhancement of the efficacy and synergy with ICIs, achieving CR in cold tumor models that are resistant to ICIs, granting a solid immune memory after treatment.

Nano‐IL‐12 profoundly activated the TME by eliciting strong secretion of inflammatory cytokines, enhanced infiltration of effector cells, and decreased presence of immunosuppressive cells. Moreover, Nano‐IL‐12 upregulated the levels of PD‐L1 on tumor cells, which can promote the activity of anti‐PD‐1 antibodies. The changes in the TME caused by Nano‐IL‐12 cooperated with anti‐PD‐1 antibodies to stimulate cancer immunity through the enhancement of antigen presentation, the increased presence of effector cells and the strengthening of their activity, and the promotion of immune cell interactions. Clinical studies have shown that anti‐PD1/anti‐PD‐L1 checkpoint blockade has a higher response rate in PD‐L1‐positive TNBC patients with high number of tumor‐infiltrating lymphocytes.^[^ ^51^, ^52^ ^]^ However, the overall performance of immune checkpoint blockade as a monotherapy against TNBC was not satisfactory^[^ ^53^, ^54^ ^]^ due to the generally low PD‐L1expression and the significantly heterogenous TME of TNBC^[^ ^55^, ^56^ ^]^ In fact, only PD‐L1 blockage in combination with chemotherapy (Nab‐Paclitaxel) has been approved in metastatic TNBC patients, which just gives a modest overall survival benefit in a small fraction of TNBC patients.^[^ ^57^ ^]^ Thus, the potential of Nano‐IL‐12 to override the immunosuppressive TME and boosting the PD‐L1 expression of TNBC may provide a robust alternative for enhancing the response rates of checkpoint blockade.

The enhanced safety of Nano‐IL‐12 is also a significant advantage for therapeutic application. Previous clinical studies have shown that systemically injected IL‐12 induces strong hematologic and hepatic toxicities.^[^ ^58^, ^59^ ^]^ Such side effects are mainly associated with the production of IFN‐*γ* and TNF‐*α* induced by IL‐12 treatment.^[^ ^42^, ^60^ ^]^ In our study, Nano‐IL‐12 showed significantly lower cytokine levels than free IL‐12 in blood and organs, which limited organ damage. Thus, we were able to inject the Nano‐IL‐12 several times at 10 µg IL‐12 per mouse, that is, around 500 µg kg^‐1^, which is approximately 1000‐fold higher than the maximum tolerated dose (MTD) of IL‐12 in humans, i.e., 500 ng kg^‐1^.^[^ ^61^ ^]^ Another important side effect of systemic IL‐12 therapy observed in clinical studies is the onset of an adaptive anti‐inflammatory response after a second administration of IL‐12, which leads to the reduction of the efficacy.^[^ ^20^, ^21^ ^]^ Such phenomenon has been associated with negative feedback mechanisms related to the overproduction of anti‐inflammatory IL‐10, and the declining of pro‐inflammatory cytokines like IFN‐*γ*, TNF‐*α*, and IL‐6.^[^ ^20^, ^22^ ^]^ Nano‐IL‐12 avoided the increase of IL‐10 in blood and organs after the second administration, which could be convenient for accomplishing repeated administration schedules without the declining of the antitumor effects. Notably, the treatment with Nano‐IL‐12 did not increase the IL‐10 concentration in tumors, maintaining high intratumoral levels of IFN‐*γ*, TNF‐*α*, and IL‐6.

Because IL‐12 is reckoned as one of the most powerful immunostimulatory cytokines, several approaches are being intensely examined to attenuate IL‐12‐induced toxicities and potentiate its effectiveness. Strategies using direct intratumoral injection, such as formulations based on IL‐12 and adjuvants,^[^ ^62^ ^]^ and plasmid DNA^[^ ^63^ ^]^ or messenger RNA^[^ ^64^ ^]^ encoding IL‐12 for producing the cytokine in situ, can maximize the therapeutic index.^[^ ^8^ ^]^ However, the local administration may face several limitations in the clinic, including the treatment of tumors growing in non‐injectable positions,^[^ ^65^ ^]^ operator‐dependent efficacies,^[^ ^66^ ^]^ uneven distribution of the injected drugs in the tumor,^[^ ^67^ ^]^ and leakage induced off‐targeting delivery.^[^ ^68^ ^]^ Moreover, the anticancer activity of the intratumorally injected formulations against distant metastasis largely relies on the variable efficacy of an abscopal effect,^[^ ^69^ ^]^ which has shown low occurrence rates in clinical studies^[^ ^70^, ^71^, ^72^ ^]^ and may not be able to surmount immunosuppressive signals in metastasis.^[^ ^73^ ^]^ The possibility to safely administer Nano‐IL‐12 by systemic intravenous injection, which is a standard clinical procedure, can allow predictable pharmacokinetics and has the potential for accessing all tumor sites through the blood supply. IL‐12‐based Fc‐fusion proteins^[^ ^14^ ^]^ and immunocytokines^[^ ^11^, ^12^, ^13^ ^]^ also share these advantages with Nano‐IL‐12. However, the IL‐12 component in these compounds is active systemically, which raises safety concerns. For example, long‐circulating fusion proteins of IL‐12 have shown high levels of serum IFN‐*γ* ^[^ ^74^, ^75^ ^]^ and IL‐12 immunocytokines have shown accumulations to off‐target site.^[^ ^76^, ^77^ ^]^ The ability of Nano‐IL‐12 to control the spatiotemporal activation could provide a safe and potent strategy with specificity in tumor targeting.

A limitation of our current study is that Nano‐IL‐12 is based on mouse IL‐12. Considering that the homology between mouse IL‐12 and human IL‐12 is around 60–70%, further studies would be necessary to determine the formulation of a human IL‐12‐based nanocytokine with activity and safety profiles that are comparable to the mouse‐based system. Auspiciously, preliminary tests have shown that the polymers used in our study can also coat human IL‐12, with similar pH‐sensitivity. Moreover, as the polymers can be precisely engineered, it would be possible to develop human IL‐12‐based system with suitable spatiotemporal profiles for human use.

In conclusion, the Nano‐IL‐12 as nanocytokines achieved effective spatiotemporal control of IL‐12 activity upon systemic administration, ensuring precise stimulation of intratumoral immunity to get satisfactory safety and therapeutic outcomes in primary and metastatic cold tumor models, synergizing with checkpoint blockade. As the polymers used for assembling Nano‐IL‐12 could be engineered for encapsulating a wide range of proteins with different molecular weight and surface charge, the system has potential for broader application prospects, such as encapsulating other therapeutic cytokines or even cytokine cocktails. Moreover, given the polymeric system could be further engineered to sense other stimuli besides pH,^[^ ^78^ ^]^ the nanocytokines can be designed for targeting various tumor microenvironments. Finally, considering the translational potential of the employed polymers and nanocytokines, this strategy holds promise for future of cancer immunotherapy.

## 4 Experimental Section

### Materials

*α*‐methoxy‐*ω*‐amino‐poly(ethylene glycol) (MeO‐PEG‐NH_2_; *M*w = 12 000 g mol^−1^) and N‐trifluoroacetyl‐L‐lysine N‐carboxyanhydride (Lys(TFA)‐NCA) were purchased from NOF Corporation

(Tokyo, Japan). Oxalyl chloride, 2‐propionic‐3‐methylmaleic anhydride (CDM), and dichloromethane (CH_2_Cl_2_) were purchased from Tokyo Chemical Industry Co. Ltd. (Tokyo, Japan). Murine interleukin‐12 (IL‐12) was purchased from GenScript (Piscataway, USA). Alexa Fluor 647 (A647) NHS ester (succinimidyl ester), ELISA kits, 2‐mercaptoethanol, Hoechst33342 solution, Halt Proteinase Inhibitor Cocktail, tissue protein extraction buffer, True‐Nuclear Transcription Factor Buffer Set, Alexa Fluor 647‐conjugated anti‐CD8*α* (anti‐CD8‐A647), Alexa Fluor 647‐conjugated anti‐T_bet_ (anti‐T_bet_‐A647), Alexa Fluor 647‐conjugated anti‐PD‐L1 (anti‐PD‐L1‐A647) were purchased from Thermo Fisher Scientific (Waltham, USA). Trehalose, DMEM medium, RPMI 1640 (RPMI) medium, penicillin–streptomycin (× 100), and L‐glutamine were purchased from Fujifilm (Tokyo, Japan). Fetal bovine serum (FBS) was purchased from Dainippon Sumitomo Pharma Co. Ltd. (Osaka, Japan). Antimouse CD4, antimouse CD8*α*, antimouse PD‐1, and antimouse CTLA4 were purchased from Bioxcell (Lebanon, USA). FITC‐conjugated anti‐Granzyme B (anti‐Granzyme B) and antimouse Asialo GM1 were purchased from Biolegend (San Diego, USA). H&E staining kit was purchased from Abcam (Cambridge, UK). RNeasy Mini Kit was purchased from Qiagen (Venlo, Netherlands). For the flow cytometry experiment conducted for 4T1‐HA tumors, the fluorescent dye and fluorescence‐labeled antibodies used were summarized in Table S3 (Supporting Information).

### Mice and Cell Lines

Female C57BL/6 mice and female BALB/c mice were purchased from Charles River Laboratory. B16F10, 4T1 cell lines were purchased from Cell Bank, Riken BioResource Center and cultured according to the instructions of ATCC. 4T1‐HA cell line was provided by Kazuyoshi Takeda (Juntendo University, Tokyo, Japan). Isolation and culture condition of murine splenocytes will be described in the following context. All animal experiments in this research were approved by The University of Tokyo and conducted under the Guidelines for the Care and Use of Laboratory Animals.

### Synthesis of CDM‐Modified Poly(ethylene glycol)‐Poly(L‐lysine)

CDM‐modified poly(ethylene glycol)‐poly(L‐Lysine) (PEG‐pLL(CDM) was synthesized via the reported method.^[^ ^27^ ^]^ First, poly(ethylene glycol)‐poly(L‐Lysine) (PEG‐pLL) was synthesized by aqueous NCA ring‐opening polymerization. MeO‐PEG‐NH_2_ (*M*w = 12 000, 500 mg) was dissolved in 10 mL 50 × 10^‐3^ m NaHCO_3_ solution (pH = 8.4). Lys(TFA)‐NCA (500 mg) was added to the ice‐bathed MeO‐PEG‐NH_2_ solution with fast stirring. The mixture was kept reacting in ice bath for 12 h, followed by dialysis (membrane MWCO = 6000–8000) against pure water, and then lyophilized. The lyophilized powder was redissolved in methanol and precipitated against diethyl ether to get purified poly(ethylene glycol)‐poly(trifluoroacetyl‐L‐Lysine) (PEG‐pLL(TFA)). The product was characterized by ^1^H NMR in DMSO‐d_6_ and GPC (TOSOH HLC‐8220 system, TOSOH, Japan; column: TSK gel G4000H_HR_; mobile phase: DMF; flow rate: 0.75 mL min^‐1^; detector: UV 220 nm) to confirm the successful polymerization. The PEG‐pLL(TFA) was dissolved in methanol containing 1 n NaOH for 12 h reaction to remove the TFA group. The mixture was purified by dialysis (membrane MWCO = 6000–8000) against pure water. After lyophilization, PEG‐pLL was obtained as a white solid. The PEG‐pLL was characterized by ^1^H NMR in D_2_O and HPLC (JASCXO LC‐EXTREMA, JASCO, Japan; column: Superdex 200‐10/300GL; mobile phase: pH 7.4 D‐PBS; flow rate: 0.75 mL min^‐1^; detector: UV 220 nm). Next, carboxydimethylmaleic anhydride (CDM) (100 mg) was dissolved in 8 mL CH_2_Cl_2_ and added with 2 mL oxalyl chloride for 12 h reaction. The mixture was dried under vacuum for removing exceed oxalyl chloride and CH_2_Cl_2_ to get the oily CDM‐Cl. CDM‐Cl was then redissolved in 2 mL CH_2_Cl_2_ and added with 8 mL CH_2_Cl_2_ containing PEG‐pLL (100 mg) for 12 h reaction. The final product, PEG‐pLL(CDM) was collected by precipitating the mixture against diethyl ether. PEG‐pLL(CDM) was characterized by ^1^H NMR in DMSO‐d_6_ and HPLC (JASCXO LC‐EXTREMA, JASCO, Japan; column: Superdex 200‐10/300GL; mobile phase: pH 3.3 acetate buffered saline with 10 × 10^‐3^ m acetate and 500 × 10^‐3^ m NaCl; flow rate: 0.75 mL min^‐1^; detector: UV 220 nm).

### Preparation and Characterization of Nano‐IL‐12

The Nano‐IL‐12 was prepared by pH‐controlled titration. PEG‐pLL(CDM) (10 mg mL^‐1^) was dissolved in pH 5.0 phosphate buffer (20 × 10^‐3^ m) to prevent the crosslinking between the CDM moieties and amino groups on the polymer chains. IL‐12 (40 µg mL^‐1^) was dissolved in pH 8.0 phosphate buffer (20 × 10^‐3^ m). For preparing Nano‐IL‐12, 200:1 molar ratio between PEG‐pLL(CDM) and IL‐12 was used. The PEG‐pLL(CDM) solution was added to the IL‐12 solution under a strictly controlled rate (2 µL min^‐1^) using a syringe pump. The mixture was then titrated to pH 7.4 by adding phosphate buffer, followed by overnight incubation with continuous shaking under 4 °C. To determine the encapsulation efficiency, A647‐labeled IL‐12 was used to prepare the Nano‐IL‐12, and the titrated mixture was loaded to HPLC (JASCXO LC‐EXTREMA, JASCO, Japan) for characterization (column: Superdex 200‐10/300GL, GE Healthcare, USA; mobile phase: pH 7.4 PBS; flow rate: 0.75 mL min^‐1^; detector: fluorescence 650/665 nm). The encapsulation efficiency was determined by the area ratio between the peak corresponding to Nano‐IL‐12 and unencapsulated free IL‐12. In the case of non‐labeled IL‐12, the encapsulation efficiency was determined by ELISA measurement for detecting the unencapsulated IL‐12 in the mixture. The Nano‐IL‐12 were purified by centrifugal infiltration using centrifugation filter (300 000 MWCO) to clear the unencapsulated IL‐12 and polymers. The size distribution of Nano‐IL‐12 was characterized by DLS and the surface charge was determined by *ζ*‐potential measurement (Zetasizer Nano‐ZS, Malvern, UK). For TEM imaging, the Nano‐IL‐12 was firstly dialyzed (300 000 MWCO) against DI water overnight to remove the phosphate salt and NaCl, then 1000 × diluted and stained by uranyl acetate and placed on 400‐mesh copper grids for TEM observation (JEM‐1400, JEOL). The formulation of the Nano‐IL‐12 was also investigated by fluorescence correlation spectroscopy (FCS). The Nano‐IL‐12 loading A647‐labeled IL‐12 was diluted in PBS to 50 × 10^‐9^ m A647 equivalence. The diluent was then added into eight‐well chamber and scanned by the FCS model of confocal laser scanning microscopy (LSM‐780, Zeiss) equipped with 633 nm excitation laser. For reference, free A647‐labeled IL‐12 was measured under the same setting.

### pH Sensitivity and Dissociation Kinetics of Nano‐IL‐12

For determining the pH sensitivity of Nano‐IL‐12, A647‐labeled IL‐12‐based Nano‐IL‐12 were prepared and concentrated to 100 µg mL^‐1^ IL‐12 equivalence in pH 7.4 PBS. The Nano‐IL‐12 solution was then 10 × diluted into PBS buffer with different pH (4.5, 5.0, 5.5, 6.0, 6.5, 7.0, 7.5, and 8.0). Upon 24 h incubation, samples were added into eight‐well chamber and scanned by the FCS mode of CLSM equipped with 633 nm excitation laser. The average diffusion coefficient of each sample was calculated.

For determining the dissociation kinetics and stability of Nano‐IL‐12, A647‐labeled IL‐12‐based Nano‐IL‐12 were prepared and concentrated to 100 µg mL^‐1^ IL‐12 equivalence in pH 7.4 PBS. The Nano‐IL‐12 solution was then 10 × diluted into PBS buffer with pH 7.4 or 6.5. At determined time points, samples were added into eight‐well chamber and scanned by the FCS mode of the CLSM using the 633 nm excitation laser. The average diffusion coefficient of each sample was calculated.

### In Vitro Bioactivity Assay

In the in vitro bioactivity assay, splenocytes collected from BALB/c mice (female, 9 weeks old) were seeded to 96‐well plates (10^5^ cells per well) in 100 µL RPMI medium (completed with 10% FBS, 1 × penicillin‐streptomycin, 2 × 10^‐3^ m L‐glutamine and 50 × 10^‐6^ m 2‐mercaptoethanol) per well. For preparing activated Nano‐IL‐12, Nano‐IL‐12 solution (100 µg mL^‐1^ IL‐12 equivalence) was first 10 × diluted in pH 6.5 PBS with overnight incubation under 4 °C, then further diluted in pH7.4 PBS containing 1%BSA to adjust the concentration for the following experiment. The splenocytes were incubated with medium containing either native IL‐12, activated Nano‐IL‐12 or nonactivated Nano‐IL‐12 (0 to 100 ng mL^‐1^ based on IL‐12 concentration) for 6 h. After this 6 h incubation, the medium containing the samples were discarded and replaced by fresh RPMI medium. The cells were cultured in the fresh medium for more 24 h. The supernatants of the cell culture medium were collected after 500 *g* × 5 min centrifugation, and the IFN‐*γ* concentrations in the samples were measured by ELISA kit following the instruction of the manufacturer.

### Preparation and Characterization of Lyophilized Nano‐IL‐12

For preparing a lyophilized formulation of Nano‐IL‐12, the Nano‐IL‐12 was first prepared and purified following the abovementioned protocol. The purified Nano‐IL‐12 solution was mixed with trehalose (5% total weight of the Nano‐IL‐12 solution) as a lyophilization protectant. The mixture was frozen by liquid N_2_ and lyophilized. For reconstitution, the lyophilized powder was reconstituted with milliQ water to the original solution volume and then kept under 4 °C for 1 h. The reconstituted Nano‐IL‐12 were first characterized by HPLC to determine the leakage rate of IL‐12 cargo, then loaded to Zetasizer for measuring the size distribution and the *ζ*‐potential.

The pH‐sensitivity of the lyophilized Nano‐IL‐12 was then determined by FCS measurement following the same protocol mentioned for checking the pH‐sensitivity of original Nano‐IL‐12, where the Nano‐IL‐12 were incubated in PBS with different pH (4.5, 5.0, 5.5, 6.0, 6.5, 7.0, 7.5, and 8.0) for 24 h, then loaded for FCS scanning.

To determine the bioactivity of lyophilized Nano‐IL‐12, in vitro splenocytes assay was used as described above. Reconstituted lyophilized Nano‐IL‐12 and activated lyophilized Nano‐IL‐12 were prepared following the protocol described in the former context. Fresh Nano‐IL‐12 were prepared for comparison. Samples were diluted to 1 ng mL^‐1^ IL‐12 equivalence in medium and incubated with the splenocytes for 24 h. The supernatants were collected for ELISA measurement to determine the IFN‐*γ* concentrations.

### Stability of IL‐12 in Plasma

The stability of IL‐12 in plasma was evaluated by SEC. Plasma was collected from a 9 weeks old BALB/c mouse with a heparinized tube, followed by centrifugation at 1000 *g* for 15 min. A647‐labeled IL‐12 was dissolved in the plasma (10 µg mL^‐1^) and incubated at 37 °C with continuous shaking. At determined incubation time points, the samples were measured by SEC (JASCXO LC‐EXTREMA, JASCO, Japan; column: Superdex 200‐10/300GL; mobile phase: pH 7.4 D‐PBS; flow rate: 0.75 mL min^‐1^; detector: fluorescence 650/665 nm).

### Pharmacokinetics of IL‐12 and Nano‐IL‐12

The blood circulation and biodistribution of Nano‐IL‐12 were investigated in mouse model. All the experiments were conducted when the average tumor volume reached 200 mm^3^. To visualize the circulation profile of Nano‐IL‐12, Nano‐IL‐12 loading A647‐labeled IL‐12 (10 µg IL‐12 equivalence) were i.v. injected to C57BL/6J mice bearing B16F10 melanoma. The earlobe skin of the mice was observed by in vivo confocal laser scanning microscopy (IVCLSM) (A1R confocal LSM, Nikon, Japan) continuously for 5 h, and the melanoma tumors were excised after 24 h for fluorescent imaging by in vivo imaging system (IVIS Spectrum, PerkinElmer, USA). To analyze the IVCLSM result, ROIs were placed in the tissue interstitium area, and the mean fluorescence intensities in the ROIs were measured at 5 h after injection. The intensity values were normalized to the maximum intensity in the vasculature immediately after injection (*V*_max_). To obtain quantitative blood circulation profile, 10 µg IL‐12 or equivalent Nano‐IL‐12 were i.v. injected to mice bearing 4T1 TNBC tumor. At determined time points, 20 µL blood sample was harvested from orbital vein and collected in a heparinized tube. Plasma samples were separated after 1000 *g* × 15 min centrifugation. For detecting the activated Nano‐IL‐12, the plasma samples were diluted in pH 7.4 PBS directly for ELISA measurement. For detecting the total amount of Nano‐IL‐12, the plasma samples were first 5 × diluted in pH 5.0 PBS and incubated for 1 h at 37 °C to completely release the encapsulated IL‐12 and then diluted in ELISA assay buffer for measurement. The mice were then sacrificed 24 h and 48 h postinjection and tissue samples were excised and homogenized by multibeads homogenizer in D‐PBS supplemented with proteinase inhibitor. After 1500 *g* × 10 min centrifugation, supernatants were collected from the tissue homogenates. The samples were first 5 × diluted in pH 5.0 PBS and incubated for 1 h at 37 °C, then diluted in ELISA assay buffer for ELISA measurement.

### Antitumor Efficacy of Nano‐IL‐12 in Primary Melanoma and Triple‐Negative Breast Cancer Models

The antitumor efficacy of Nano‐IL‐12 was evaluated in primary melanoma and triple‐negative breast cancer (TNBC) tumor models. To establish the melanoma model, C57BL/6J mice (5 weeks) were inoculated with B16F10 cells (10 ^6^ cells suspended in 100 µL DMEM medium per mouse) subcutaneously at the left abdomen. To establish the orthotopic breast cancer model, BALB/c mice (5 weeks) were inoculated with 4T1 cells (10^6^ cells suspended in 100 µL RPMI medium per mouse) at the left mammary fat pad. The tumor volume and body weight of the mice were tracked continuously during the experiments, where the tumor volume was calculated by the formula bellow:

(1) $$V=\frac{1}{2}L\times W^{2}$$

where *V* is the volume of the tumor, *L* is the length and *W* is the width. *L* and *W* were measured by caliper scale. All the treatments were initiated on randomly grouped mice when the average tumor volume reached 30 mm^3^. The specific treatment schedules and doses were described in the corresponding figures. For intravenous (i.v.) routes, the compounds (PBS, IL‐12 and Nano‐IL‐12) were injected in a volume of 200 µL. For intraperitoneal (i.p.) routes, the compounds (anti‐PD1 and anti‐CTLA4 antibodies) were administered in a volume of 100 µL. Mice were euthanized when the tumor exceeded 1000 mm^3^ and/or based on humane end‐point criteria.

### Systemic and Intratumoral Cytokine Analysis

Mice bearing 4T1 TNBC tumor (average tumor volume: 200 mm^3^) were i.v. injected twice with 10 µg IL‐12 or equivalent Nano‐IL‐12 on Day 0 and Day 3. Then, the mice were divided into 2 groups. One group of the mice was used for blood sampling, where 100 µL blood was collected from the orbital vein every day and the plasma samples were collected after centrifugation at 1000 g for 15 min. The other group of mice were used for tissue analysis, where the mice were sacrificed on Days 2 and 5, and the organs and tumors were collected. Tissue samples were homogenized by multibeads homogenizer in tissue protein extraction buffer supplemented with proteinase inhibitor and the supernatants were collected after centrifugation at 10 000 *g* for 10 min. The concentrations of cytokines (IFN‐*γ*, TNF‐*α*, IL‐6, and IL‐10) in plasma and tissue samples were determined by ELISA kit.

### Evaluation of Systemic Toxicity

Healthy female BALB/c mice (5 weeks old) were i.v. injected twice with 10 µg IL‐12 or equivalent Nano‐IL‐12 on Day 0 and Day 3 with daily recording the bodyweight. On Day 7, mice were sacrificed for harvesting blood, liver, and kidney samples. The blood samples were kept under room temperature for complete coagulation, followed by centrifugation at 4000 *g* for 10 min to collect serum. The serum samples were loaded to blood chemistry analyzer (DRI‐CHEM NX700, Fujifilm) to detect the concentration of total protein (TP), alanine aminotransferase (ALT), blood urea nitrogen (BUN), lipase and creatinine (CRE). The liver and kidney samples were embedded in O.C.T compound and frozen by liquid N_2_. The tissues were sliced by cryostat (CM1950, Leica) to 10 µm thickness sections. The sections were stained by H&E kit and observed by microscope (BZ‐X810, Keyence) with brightness field.

### Flow Cytometry Analysis of Intratumoral CTLs in Melanoma

For lymphocyte infiltration analysis, the experiments started when the average tumor size reached to 100 mm^3^ (8 d after inoculation). The mice were i.v. injected twice on 8 and 11 d postinoculation respectively, with PBS, 1 µg IL‐12, or equivalent Nano‐IL‐12 in 200 µL solution. Tumors were harvested 7 d after the final injection. The tumors were digested by DMEM containing 1 mg mL^‐1^ collagenase for preparing the single‐cell suspension. The cells were stained by FITC‐labeled anti‐CD3‐FITC and A647‐labeled anti‐CD8*α* antibodies in PBS containing 2% FBS. Then, the samples were analyzed by flow cytometer (BD LSR II, BD Biosciences, USA). Lymphocytes were firstly gated out from all cells via FSC‐SSC plotting, and then plot against FITC‐APC channel for analysis of the subtypes.

### Effect of Immune Cell on Antitumor Activity

BALB/c mice (female; 5 weeks old) were orthotopically inoculated with 10^6^ 4T1 cells on Day 0. On Days 7, 9, 11, 13, and 15, the mice in treatment groups were i.v. injected with 1 µg IL‐12 equivalent Nano‐IL‐12 dispersed in 200 µL PBS. On Days 6, 8, and 10, the mice in corresponding groups were i.p. injected with 200 µg anti‐CD4 (for depleting CD4^+^ cells), anti‐CD8 (for depleting CD8^+^ cells), anti‐asialo GM1 (for depleting NK cells) dispersed in 100 µL PBS respectively. The tumor volume was tracked continuously during the experiment. Mice were euthanized when the tumor exceeded 1000 mm^3^ and/or based on humane end‐point criteria.

### Immunohistological Analysis

Mice bearing orthotopic 4T1 TNBC tumors (average tumor volume: 200 mm^3^) were i.v. injected twice with 10 µg IL‐12 or equivalent Nano‐IL‐12 on Day 0 and Day 3. On Day 7, the mice were sacrificed for collecting the tumors. Tumor samples were embedded in O.C.T compound and frozen by liquid N_2_, then sliced by cryostat (CM1950, Leica) into 10 µm thick sections. The sections were separately stained by H&E kit or by antibodies for immunohistochemistry. The sections for immunofluorescence staining were stained by Hoechst33342. Then, to stain CD8 and PD‐L1, the sections were blocked by 1% BSA solution overnight and then incubated with A647‐labeled anti‐CD8*α* or anti‐PD‐L1 antibodies diluted according to the instruction from the manufacturer under room temperature for 1 h. To stain T_bet_ and Granzyme B, the sections were permeabilized by transcription factor staining buffer set, then blocked by 1% BSA solution overnight, and incubated with A647‐labeled anti‐T_bet_‐A647 or FITC‐labeled anti‐Granzyme B antibody according to the instruction from the manufacturer at room temperature for 1 h. For H&E, CD8, T_bet_ and PD‐L1 staining, the sections were observed by microscope (BZ‐X810, Keyence) with brightness field for H&E, or DAPI and Cy5 fluorescence channels for immunofluorescence staining. For Granzyme B/CD8 double staining, the sections were observed by confocal microscope (LSM780, Zeiss) equipped with 405, 488, and 633 nm lasers (emission filters: 460, 510, and 660 nm, respectively).

### Flow Cytometry Analysis and RNA Sequencing of 4T1‐HA Tumors

BALB/c mice (female; 5 weeks old) were orthotopically inoculated with 10^6^ 4T1‐HA cells on Day 0. On Days 7 and 9, the mice were i.v. injected with 10 µg IL‐12 or equivalent Nano‐IL‐12 in 100 µL solution. On Days 8 and 10, the mice in corresponding groups were i.p. injected with 100 µg anti‐PD‐1 antibodies in 100 µL PBS. Tumor samples were harvested on Day 17. Each tumor sample was divided into two parts: one for flow cytometry analysis and the other for RNA sequencing (RNA‐seq). For flow cytometry analysis, the tumors were digested by RPMI containing 2 mg mL^‐1^ collagenase with 0.02 mg mL^‐1^ DNase for preparing the single‐cell suspension. The cells were stained with corresponding antibody panels (Table S4, Supporting Information). For staining the membrane targets, the cells were incubated with the antibodies dissolved in 2% FBS containing PBS. For staining the intracellular targets, cells were permeabilized by transcription factor staining buffer set, then stained with antibodies dissolved in 2% FBS containing PBS. The samples were then loaded to flow cytometer for analysis (Gallios, Beckman Coulter, USA). The gating strategies for analyzing the cell populations were included in Figures S8–S10 (Supporting Information). RNA‐seq measurements were conducted by Veritas (Japan). Total RNA samples were isolated from tumor tissues using RNeasy spin columns according to the manufacturer's protocols. Libraries were prepared using NEBNext Ultra II RNA Library Prep Kit for Illumina (New England Biolabs, Ipswich, Massachusetts, USA) according to the manufacturer's protocols. The libraries were sequenced as 150 bp paired‐end reads using NovaSeq 6000 (Illumina). The sequence reads were aligned to the mm10 reference genome using STAR V.2.7.0f. Mapped reads were counted by featureCounts V.1.6.4. The read values were normalized to FPKM. Differentially expressed gene analysis, KEGG and GOBP enrichment were analyzed by DESeq2 package via R. Tumor‐infiltration immune and stromal cell populations were analyzed by the reported method.^[^ ^79^ ^]^ The RNA‐seq raw data have been deposited on Gene Expression Omnibus (GEO) database (accession number: GSE196030).

### Antitumor Efficacy against Metastatic TNBC Model

Orthotopic mouse model of spontaneous TNBC metastasis was established on BALB/c mice. Orthotopic primary TNBC tumors were first established following the same protocol described in the former context. After the average tumor volume exceeded 200 mm^3^, mice were anesthetized under flow of 2% isoflurane. The tumor was removed by sterile scissors and the skin of surgery area were sutured. The metastasis will then form spontaneously after the surgery. Three days after tumor resection, the treatment was started. The specific treatment schedule and dose were described in the corresponding figure. For intravenous (i.v.) routes, the compounds (saline, IL‐12, and Nano‐IL‐12) were injected in a volume of 200 µL. For intraperitoneal (i.p.) routes, the compounds (anti‐PD1 and anti‐CTLA4 antibodies) were administered in a volume of 100 µL. Eight days after tumor resection, part of the mice were sacrificed for harvesting the lung samples. The lungs were sliced and stained with H&E to recognize the metastasis. Other mice were kept for monitoring the survival percentage.

### Tumor Rechallenge Studies

Mice surviving 90 d post‐inoculation were recognized as cured mice. The cured mice were rechallenged with the same cancer cells that were originally inoculated. For the subcutaneous melanoma model, the mice were s.c. injected with 10^5^ B16F10 cells suspended in 50 µL DMEM medium at the symmetry point of the inoculation position. For the orthotopic TNBC model, the mice were injected with 10^5^ 4T1 cells suspended in 50 µL RPMI medium at the symmetry point of the inoculation position. For metastatic TNBC model, the mice were i.v. injected with 10^5^ 4T1 cells suspended in 50 µL RPMI medium.

## Conflict of Interest

P.C., G.L.H., T.M., and H.C. are inventors of a patent on Nano‐IL‐12. Dr. T.M. and H.C. are co‐founders of Red Arrow Therapeutics, which have licensed this patent. K.K. and K.K. are scientific advisors of Red Arrow Therapeutics.

## Author Contributions

P.C. performed the experiments and wrote the manuscript. W.Y., S.L., and K.I. assisted with animal experiments. K.N. and K.T. contributed to the immune analysis of 4T1‐HA tumors and helped with data analysis. G.L.H and T.M. assisted with the synthesis and characterization of the polymers used in this research. G.L.H and T.H. assisted with preparation and characterization of the nanoparticles. K.K. supervised the immune analysis. K.K. supervised the project and edited the manuscript. H.C. conceived the study, supervised the project, and edited the manuscript. All authors checked and advised the structure of the manuscript.

## Supporting information

Supporting Information

## Data Availability

The data that support the findings of this study are available from the corresponding author upon reasonable request.
