# Supplementary material for: An IL‐12‐Based Nanocytokine Safely Potentiates Anticancer Immunity through Spatiotemporal Control of Inflammation to Eradicate Advanced Cold Tumors
Source: Adv Sci (Weinh). 2023 Feb 5;10(10):2205139. doi: 10.1002/advs.202205139 (PMC10074049; doi:10.1002/advs.202205139)
Supplement: Supplementary file 1 — Supporting Information [file ADVS-10-2205139-s001.pdf]

Supplementary Information

An IL-12-based nanocytokine safely potentiates anticancer immunity through spatiotemporal control of inflammation to eradicate advanced cold tumors

Pengwen Chen, Wenqian Yang, Koji Nagaoka, George Lo Huang, Takuya Miyazaki, Taehun Hong, Shangwei Li, Kazunori Igarashi, Kazuyoshi Takeda, Kazuhiro Kakimi, Kazunori Kataoka*, Horacio Cabral**

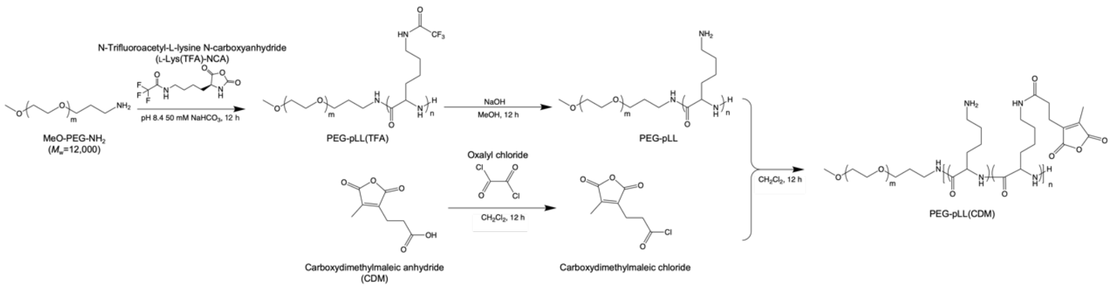

Scheme S1. Synthesis route of PEG-pLL(CDM) polymer.

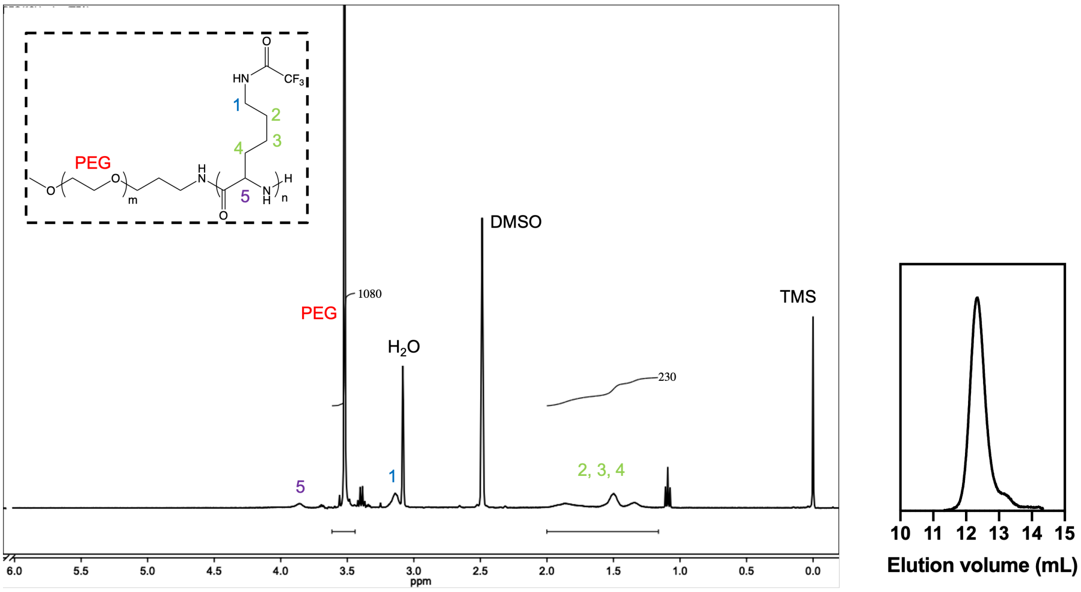

Figure S1. ^{1}H-NMR spectrum and GPC result of PEG-pLL(TFA). The degree of polymerization (DP) of lysine group was determined by comparing the integration of the peaks of -CH_2-CH_2- on PEG (δ = 3.5 ppm) and -CH_2-CH_2-CH_2- on lysine (δ = 1.2 – 2.0 ppm, peak 2, 3 and 4). The units of the pLL(TFA) block were determined to be 40.

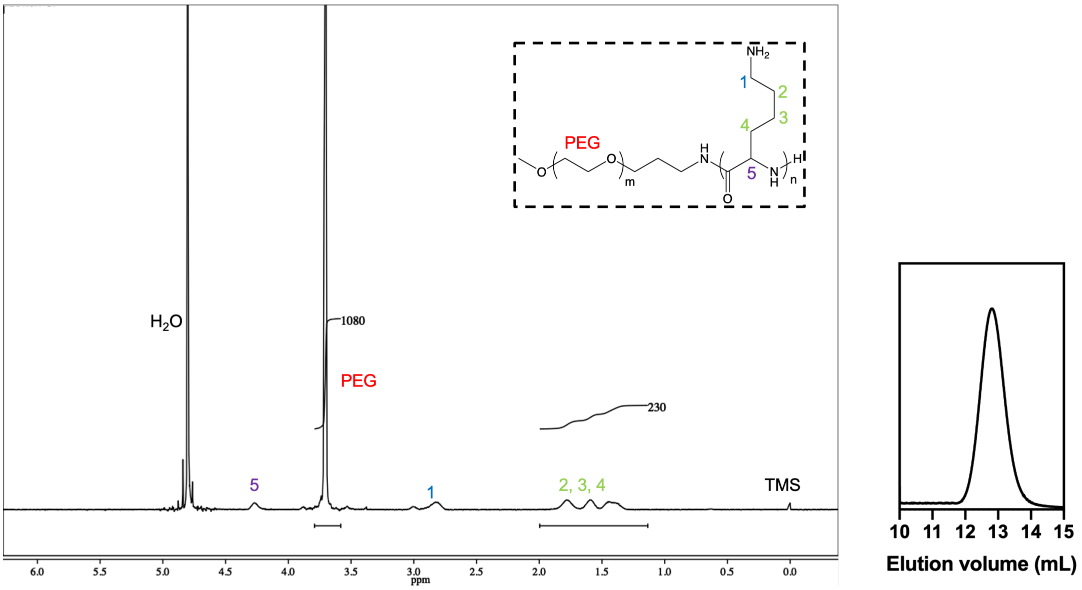

Figure S2. ^{1}H-NMR spectrum and HPLC result of PEG-pLL. The degree of polymerization (DP) of lysine group was determined by comparing the peaks of -CH_2-CH_2- on PEG (δ = 3.5 ppm) and -CH_2-CH_2-CH_2- on lysine (δ = 1.2 – 2.0 ppm, peak 2, 3 and 4). The units of the pLL block were determined to be 40.

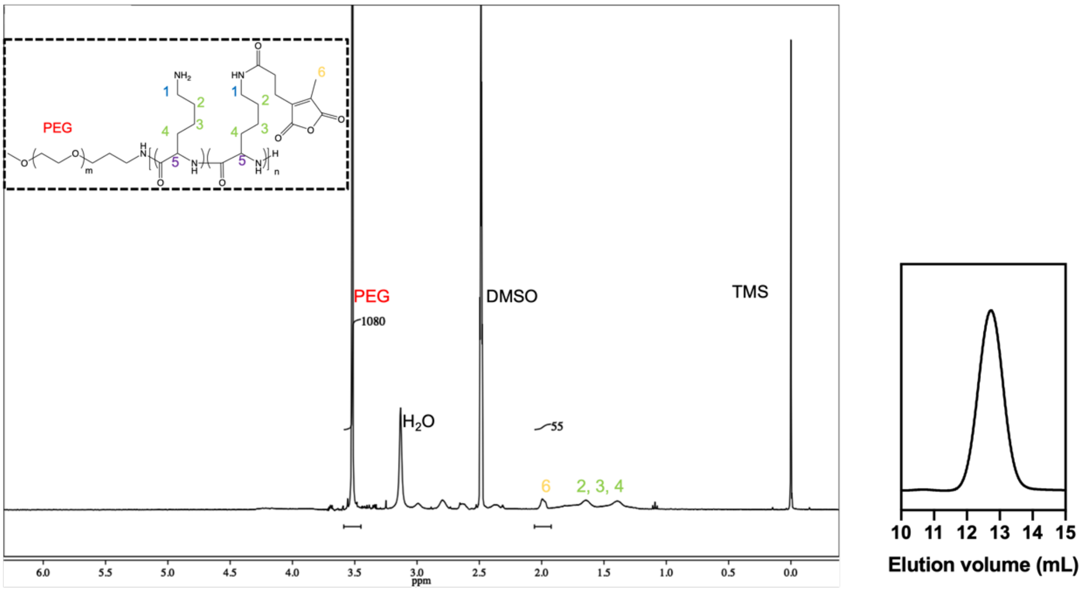

Figure S3. ^{1}H-NMR spectrum and HPLC result of PEG-pLL(CDM). The conjugation of CDM to the polymer was confirmed by the appearance of the peaks corresponding to -CH_3 on CDM ($\delta = 2.0$ ppm, peak 6). The number of CDM groups conjugated to one polymer molecule was determined to be 20 from the integration of PEG peak and peak 6.

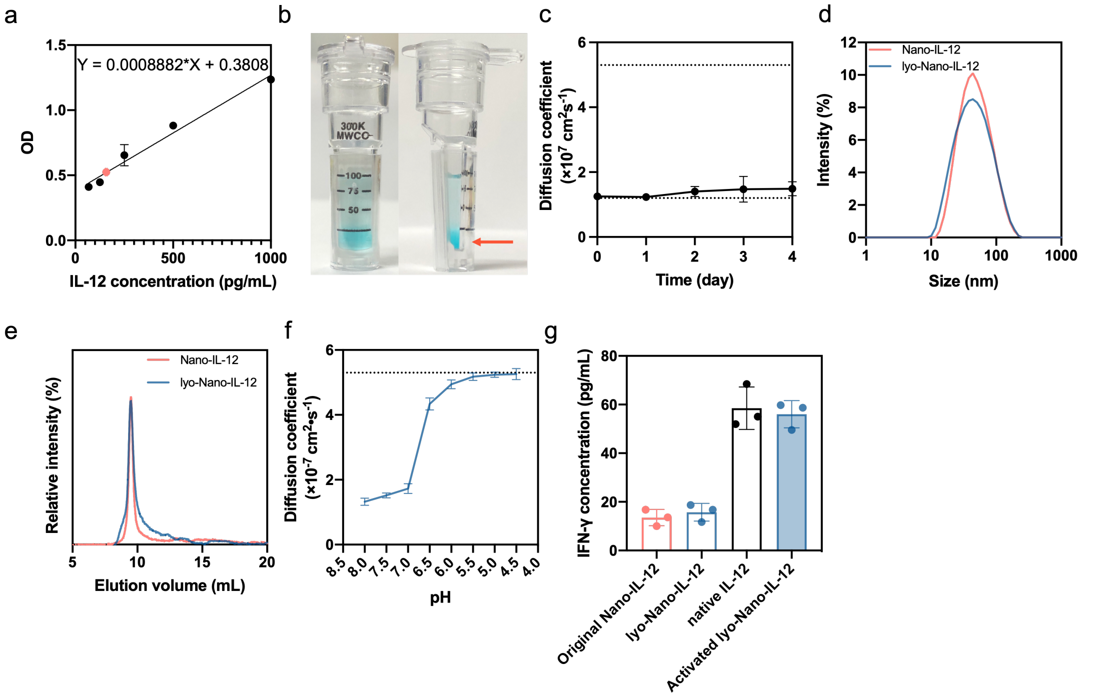

Figure S4. Characterization of original Nano-IL-12 and lyophilized Nano-IL-12. a) Representative ELISA result of the titrated mixture during preparation of the Nano-IL-12. The red dot indicates the unencapsulated IL-12 concentration in the diluted solution (corresponding concentration: 158 ± 4 pg/mL), while the total feeding amount of IL-12 in the solution should correspond to 1000 pg/mL. The result indicates that the encapsulation efficiency of IL-12 was around 80%. **b)** After centrifugal filtration through membrane with 300,000 MWCO, the A647-labelled IL-12 was retained by the membrane, suggesting the formation of macromolecular structure with a molecular weight exceeding 300,000 Da. **c)** Long-term stability profile of Nano-IL-12 under pH7.4 measured by the FCS method. The dotted lines at 1.2×10^7 cm^{2}s^{-1} and 5.3×10^7 cm^{2}s^{-1} refer to the diffusion coefficients of intact Nano-IL-12 and released free IL-12, respectively. **d)** Representative DLS result of Nano-IL-12 and lyophilized Nano-IL-12. **e)** Representative HPLC result of original Nano-IL-12 and lyophilized Nano-IL-12 loading A647-labelled IL-12. **f)** pH-sensitivity of lyophilized Nano-IL-12 indicated by FCS measurement of the Nano-IL-12 incubated in different pH condition for 24 h. The dotted line at 5.3×10^7 cm^{2}s^{-1} refers the diffusion coefficient of free IL-12. **g)** Splenocytes assay confirmed the activatable bioactivity of lyophilized Nano-IL-12 after reconstitution (Data shown as mean ± S.D.; *n* = 3 parallel measurements).

Table. S1 Fluorescence correlation spectroscopy measurement results

Sample	Counts per molecule (kHz)	Number of IL-12 in single particle	Diffusion time (μs)	Diffusion coefficient ($\times 10^{-7}$ $cm^2 \bullet s^{-1}$)	Hydrodynamic diameter (nm)[b]
A647	3.53±0.2	-	63.6±3	33 [a]	-
A647-IL-12	5.08±0.1	-	397.5±15	5.3±0.2	8.4±0.3
A647-Nano-IL-12	7.81±1.1	≈ 1.5	1812.8±14	1.2±0.1	37.2±3.1

[a] Reported result [(1)]

[b] Calculated by *Stokes-Einstein* equation

Table. S2 Characterization of original and lyophilized Nano-IL-12

Sample	average size[a] (nm)	PDI	ζ-potential (mV)[b]
IL-12	-	-	-28.5 ± 3.2
original Nano-IL-12	42 ± 2	0.18 ± 0.1	-4.1 ± 1.0
lyophilized Nano-IL-12	47 ±1	0.21 ± 0.2	-2.9 ± 0.7

[a] Determined by DLS

[b] Measured in pH 7.4 condition

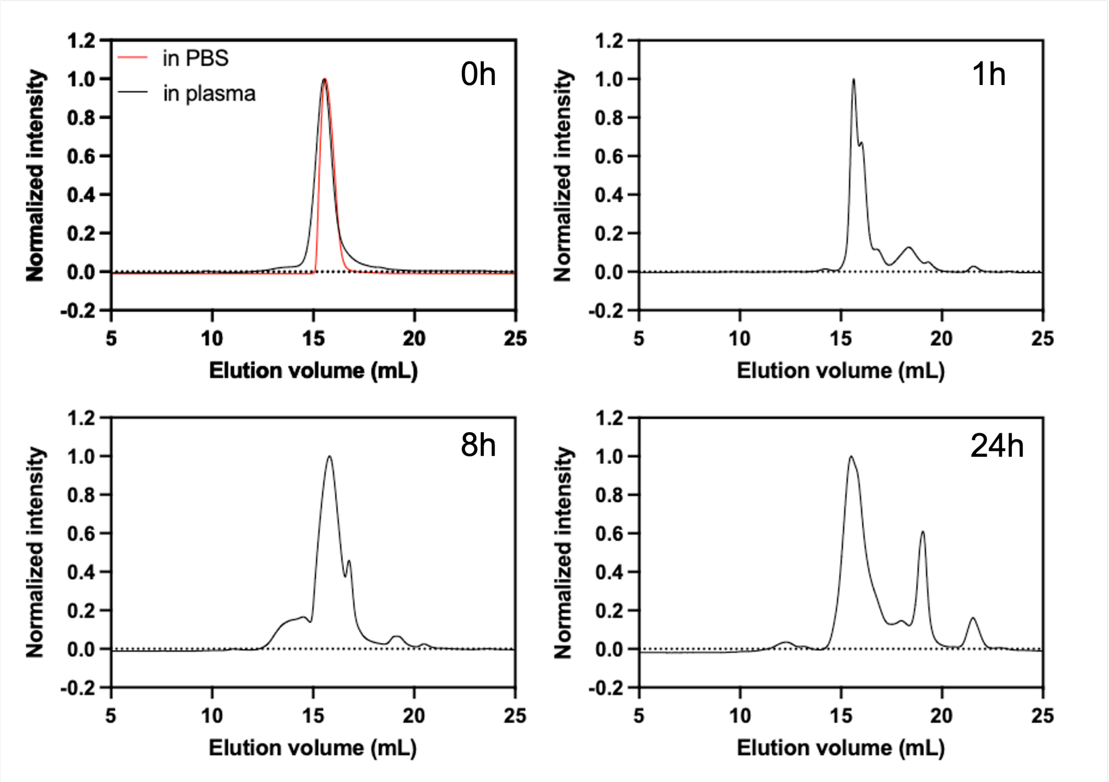

Figure S5. Stability of IL-12 in plasma. A647-labeled IL-12 was incubated in mouse plasma for determined time and studied by SEC detecting the fluorescence signal.

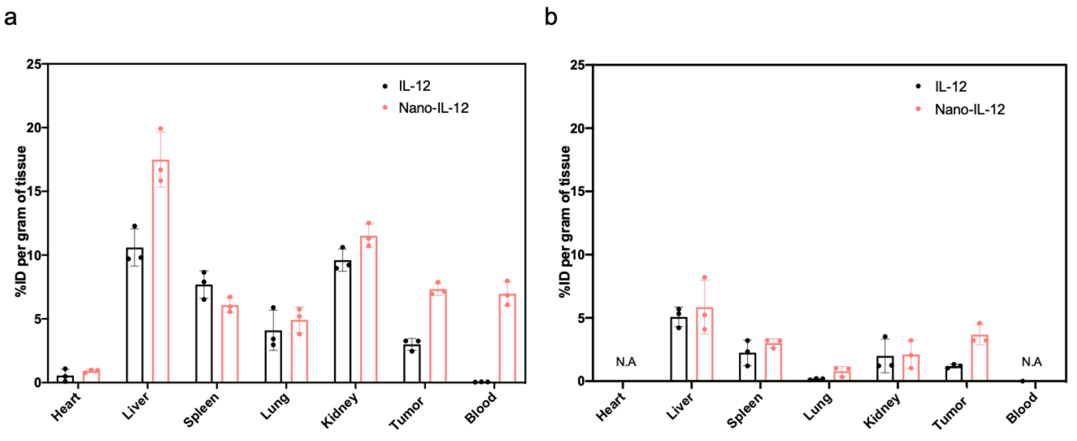

Figure S6. Biodistribution of IL-12 or Nano-IL-12 upon i.v. injection with 10 µg IL-12 or equivalent Nano-IL-12. a) Biodistribution measured by ELISA 24 h post injection. **b**) Biodistribution measured by ELISA 48 h post injection. The experiment was done in 4T1 tumor-bearing mice (Data shown as mean ± S.D.; n = 3 mice per group)

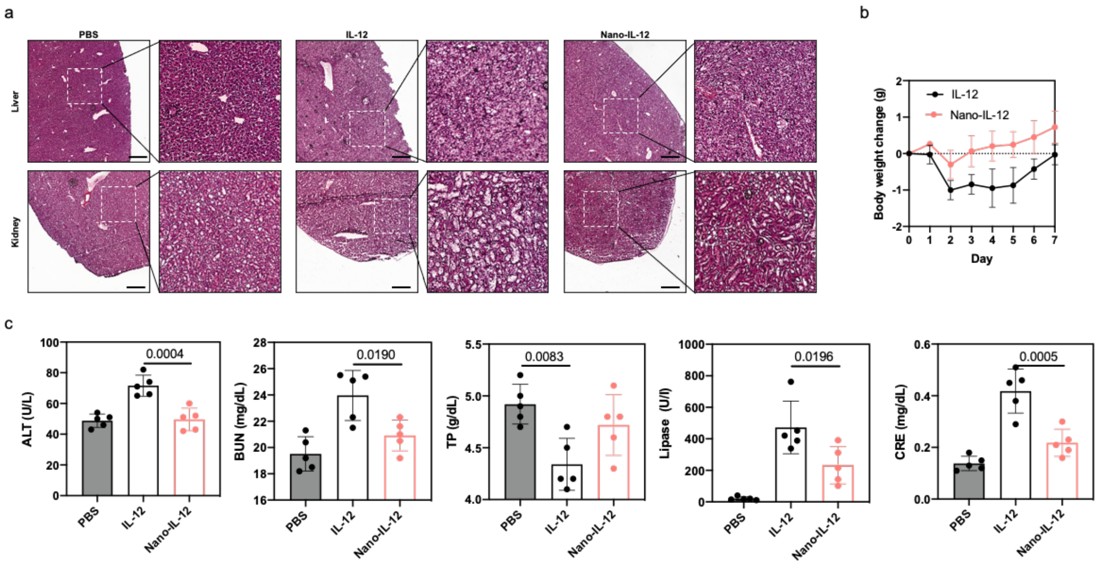

Figure S7. Evaluation of the systemic toxicity of Nano-IL-12 treatment in healthy BALB/c mice. a) Representative microscopic images of H&E-stained liver and kidney sections collected from mice sacrificed on Day 7 after two injections of 10 µg IL-12 or equivalent Nano-IL-12 on Days 0 and 3. Scale bar = 100 µm. **b**) Bodyweight record of the mice during the experiment (Data are shown as mean ± S.D.; $n = 5$ mice per group). **c**) Organ damage-associated biomarkers measured in plasma from the sacrificed mice (Data are shown as mean ± S.D.; $n = 5$ mice per group; p values are calculated *via* one-way ANOVA).

Table S3. List of the antibody and fluorescent dye used in flow cytometry analysis of 4T1-HA tumors

Category	Product name	Vendor
Antibody	anti-CD16/32 (clone 2.4G2)	Bio X Cell
Antibody	APC-conjugated anti-CD25	Thermo Fisher Scientific
Antibody	APC-conjugated anti-CD3ε	BioLegend
Antibody	APC/Cyanine7-conjugated anti-CD45	BioLegend
Antibody	APC/Cyanine7-conjugated anti-CD8	BioLegend
Antibody	FITC-conjugated anti-CD19	BioLegend
Antibody	FITC-conjugated anti-CD3ε	BioLegend
Antibody	FITC-conjugated anti-CD8 (clone KT15)	Medical & Biological Laboratories
Antibody	Pacific Blue-conjugated anti-CD45	BioLegend
Antibody	PECF594-conjugated anti-CD45R/B220	BD Biosciences
Antibody	PE/Dazzle594-conjugated anti-F4/80	BioLegend
Antibody	PE/Dazzle594-conjugated anti-Ly6G	BioLegend
Antibody	PE-conjugated anti-CD49b	BioLegend
Antibody	PE-conjugated anti-Foxp3	Thermo Fisher Scientific
Antibody	PE-conjugated Rat IgG2a isotype control	Thermo Fisher Scientific
Antibody	PE/Cyanine7-conjugated anti-CD8	BioLegend
Antibody	PE/Cyanine7-conjugated anti-PD-1	BioLegend
Antibody	PerCP/Cyanine5.5-conjugated anti-CD4	BioLegend
Cell viability dye	Zombie Yellow Fixable Viability Kit	BioLegend
Tetramer staining	T-Select H-2Kd Influenza HA Tetramer-IYSTVASSL-PE	Medical & Biological Laboratories
Tetramer staining (control)	T-Select H-2Kd EGFP Tetramer-HYLSTQSAL-PE	Medical & Biological Laboratories

Table. S4 Staining panel for flow cytometry analysis of 4T1-HA tumors

Panel	FITC	PE	PI, PECF594	PerCP-Cy5.5	PE-Cy7	APC	APC/Cy7	PB	ZY
T, B, NK	CD19	CD49b	F4/80, Ly6G	CD4	/	CD3	CD8	CD45	Zombia Yellow
HA-Tetramer/PD-1	CD8	HA-Tetramer/EGFP-Tetramer (control)	F4/80, Ly6G, B220	CD4	PD-1	CD3	/	CD45	Zombia Yellow
Treg-Foxp3	CD8	Foxp3/ Rat IgG isotype (control)	F4/80, Ly6G, B220	CD4	CD8	CD3	CD45	/	Zombia Yellow

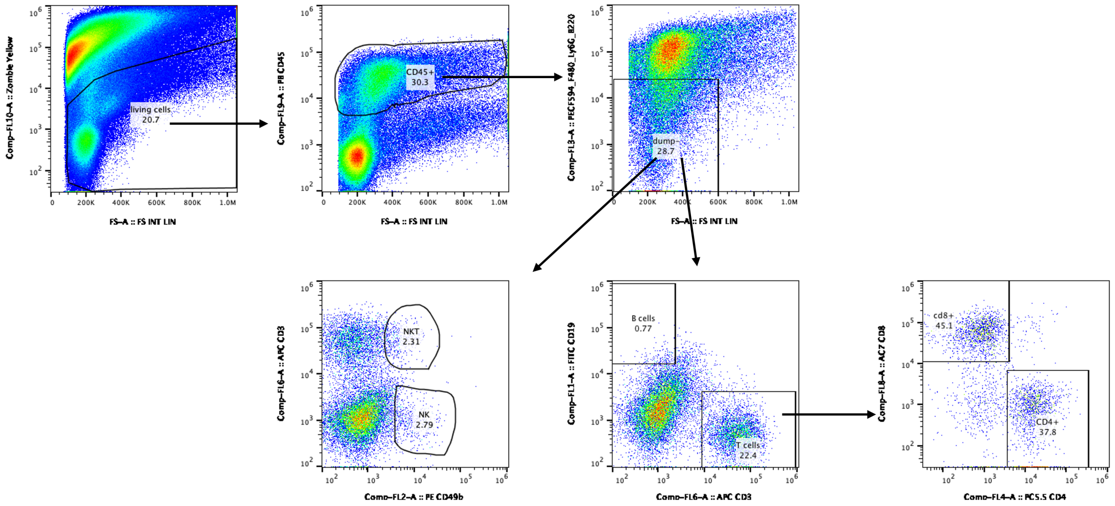

Figure S8. Gating strategy for defining the T cells, B cells and NK/NKT cells in tumors.

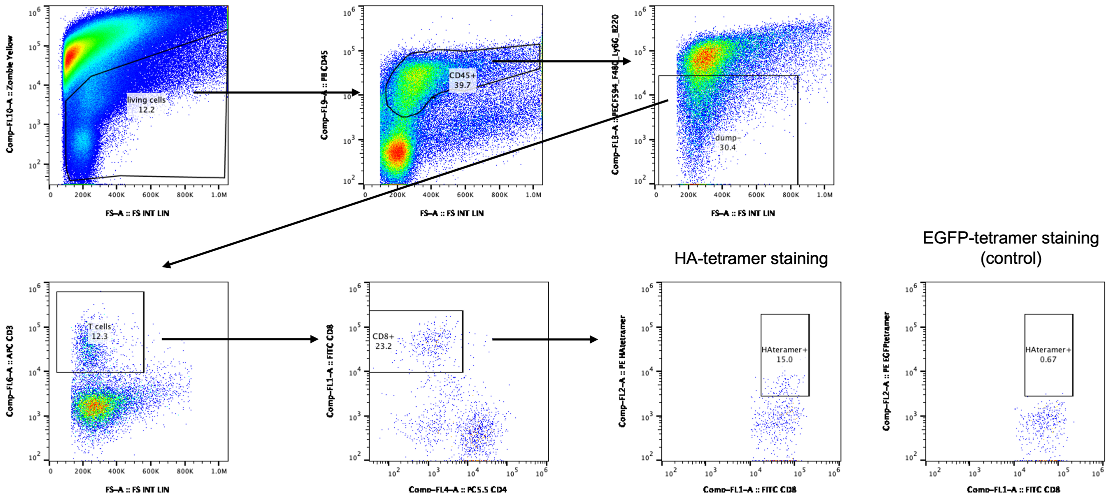

Figure S9. Gating strategy for defining the HA-tetramer positive CTLs in 4T1-HA tumors.

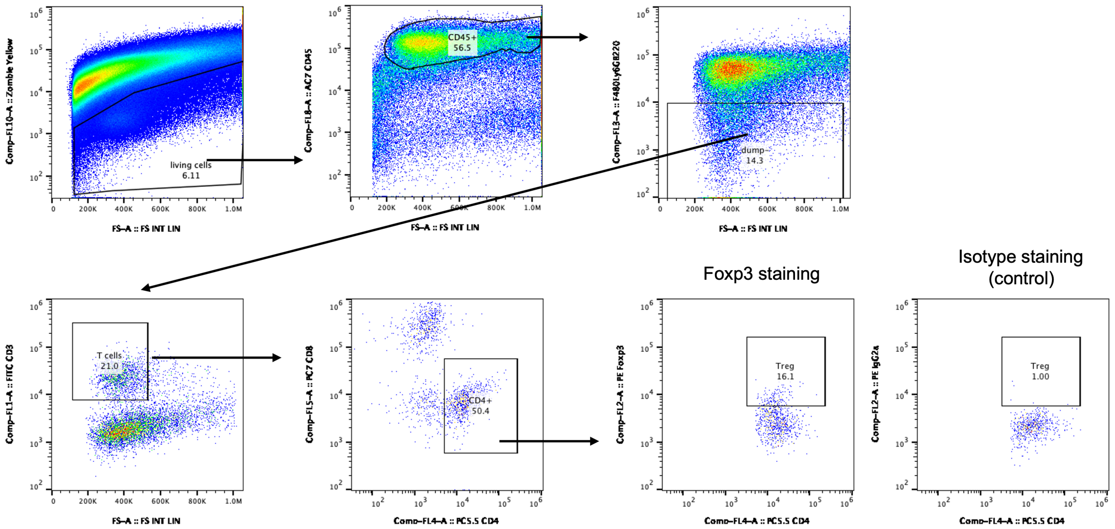

Figure S10. Gating strategy for defining the Foxp3+ Tregs in tumors.

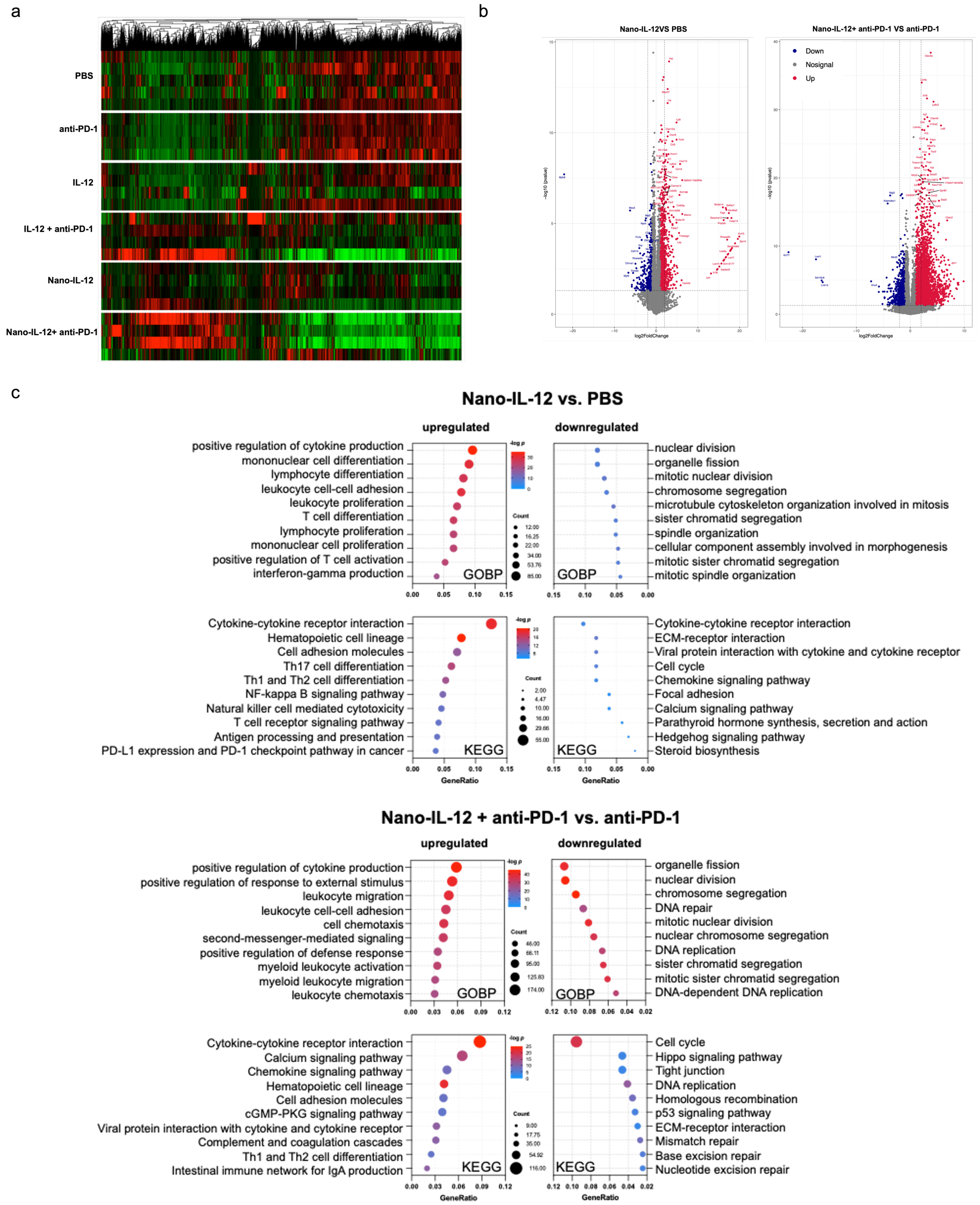

Figure S11. RNA-seq revealed profound changes in the TME after Nano-IL-12 and Nano-IL-12 plus anti-PD-1 antibodies treatment. a) Heatmap of the global gene expression in TME. Gene expression level was plotted as Z-score. **b)** Comparison analysis of the differentiated gene expression in Nano-IL-12 vs. PBS treated tumors (left panel) and Nano-IL-12 + anti-PD-1 vs. anti-PD-1 treated tumors (right panel). **c)** Enrichment analysis of the differentiated gene expression in b) revealed the upregulation of pathways associated with immune activation (left column) and the downregulation of pathways associated with cell proliferation (right column).

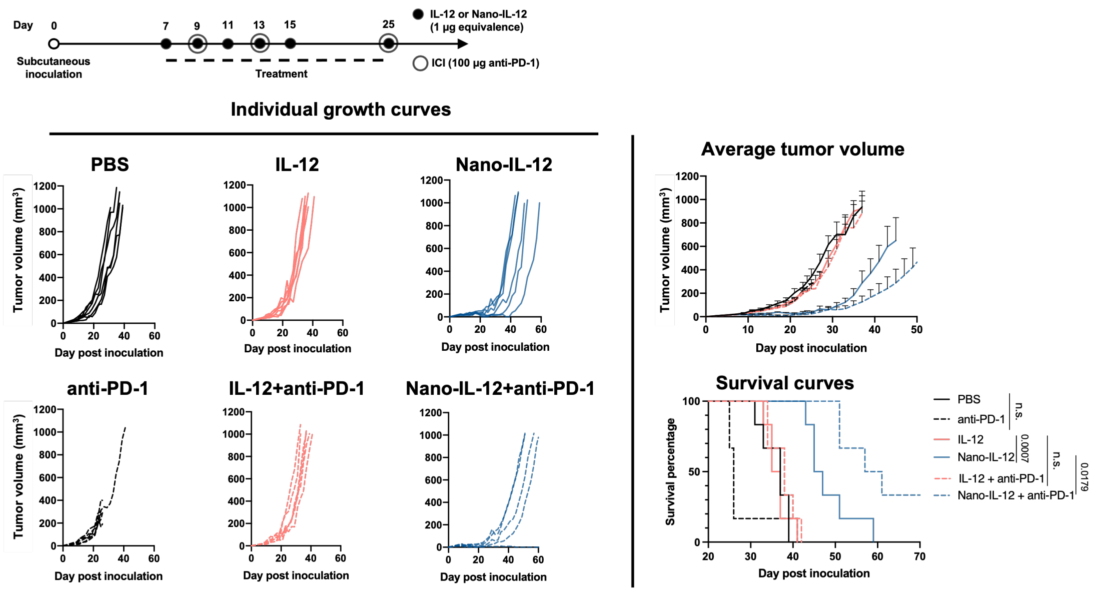

Figure S12. Repeated administration of Nano-IL-12 at 1 ug IL-12 equivalence per mouse combined with ICIs showed potent therapeutic efficacy in orthotopic TNBC tumors. Mice were orthotopically inoculated with 4T1 cells, and received PBS, IL-12, Nano-IL-12, IL-12 + ICI and Nano-IL-12 + ICI (dose and schedule are shown in the scheme presented at the upper panel). Individual tumor growth curves are shown in the left panel. Average tumor volume curves and survival rate are shown in the right panel. Nano-IL-12 + ICI strongly inhibited the progression of tumor and leading to CR (2/6). (Data shown as the mean ± SEM; n = 6 mice per group; p values calculated by log-rank analysis).

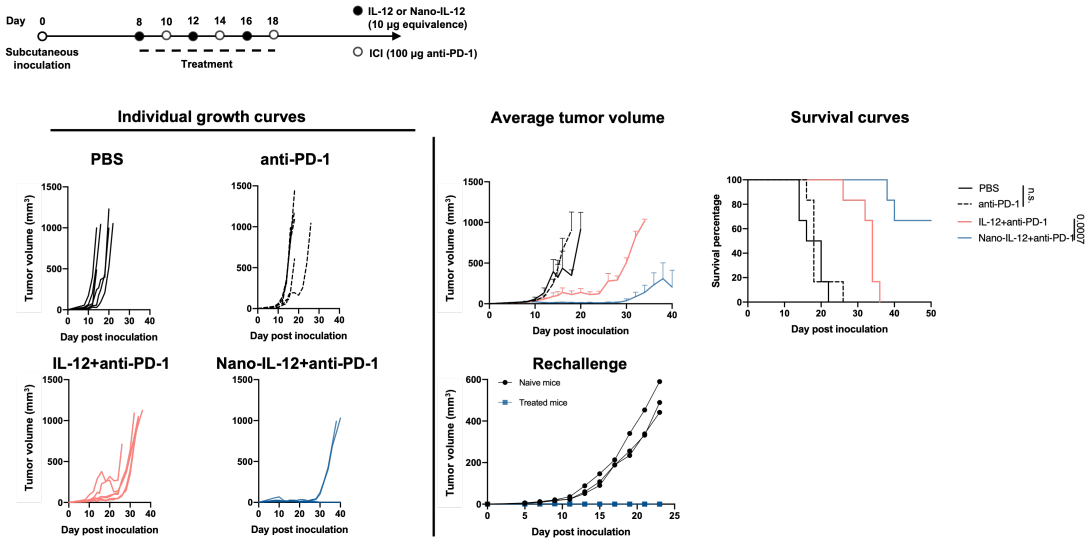

Figure S13. Combination therapy of Nano-IL-12 with ICIs leads to strong therapeutic efficacy in melanoma tumors. Mice received three times treatment with 10 µg IL-12 or equivalent Nano-IL-12, with staggered treatment of anti-PD-1 antibodies (scheme presented at the upper panel). The combination therapy of Nano-IL-12 with anti-PD-1 strongly inhibited the progression of tumor and leading to CR (4/6). Moreover, the mice surviving in the Nano-IL-12 + ICIs were rechallenged by a subcutaneous inoculation of B16F10 cells, and the tumor growth was followed for 24 days (right lower panel). The mice showed immunological memory against B16F10 cells (Data shown as the mean ± SEM; *n* = 6 mice per group; *p* values calculated by log-rank analysis).

Reference

Kapusta, P. Absolute Diffusion Coefficients: Compilation of Reference Data for FCS Calibration. *PicoQuant GmbH* **2010**, *1*, 1-2
